# A multimodal deep learning-based model for posture asymmetry recognition and sports injury risk prediction in adolescent table tennis athletes

**DOI:** 10.3389/fphys.2026.1800522

**Published:** 2026-04-01

**Authors:** Di Wang, Yue Guo

**Affiliations:** 1 School of Physical Education, Yantai University, Yantai, Shandong, China; 2 School of Physical Education, University of Jinan, Jinan, Shandong, China

**Keywords:** adolescent athletes, multimodal deep learning, posture asymmetry, sports injury prediction, table tennis

## Abstract

**Background:**

Adolescent table tennis athletes face significant sports injury risks due to repetitive unilateral force generation patterns during the critical skeletal maturation period, yet traditional posture assessment methods lack quantitative precision and real-time monitoring capability.

**Methods:**

This study develops a multimodal deep learning framework integrating video RGB sequences, skeletal keypoint trajectories, and kinematic parameters through cross-modal attention mechanisms, weighted graph convolutional networks, and temporal convolutional networks to automatically recognize posture asymmetry patterns and assess biomechanical injury risk levels based on expert-evaluated postural deviation criteria, representing prospective biomechanical risk stratification for screening purposes rather than longitudinally validated injury occurrence prediction.

**Results:**

Comprehensive evaluation on the TTStroke-21 dataset demonstrates superior performance in both four-class posture asymmetry recognition and three-level injury risk prediction compared to baseline methods, validating the effectiveness of sport-specific architectural adaptations and multimodal data fusion strategies. The biomechanical analysis reveals quantitative relationships between technical movement patterns and asymmetry manifestations across different stroke types and age groups, confirming the critical intervention window during the 12-14-year developmental period.

**Conclusion:**

The proposed intelligent assessment system provides substantial practical value for training monitoring and injury prevention in youth sports, enabling coaches and sports medicine practitioners to implement data-driven personalized intervention strategies including contralateral limb strengthening programs and targeted corrective exercises before structural imbalances progress to clinical injury outcomes.

## 1 Introduction

Teenage players of table tennis incur a special risk of injury related to sports as they go through development. Incomplete ossification of the muscular system, asymmetry of muscle strength development, and the unilateral loading associated with table tennis incur prolonged asymmetrical training. This affects the balance of posture associated with the shoulder, spinal, and pelvic areas. Research has found that there is a marked relationship between muscle strength imbalances in the shoulders and abnormal positioning of the scapulae among adolescent players (Akınoğlu et al., 2020), which is further accentuated by forehand attack exercises performed during table tennis training (Mocanu et al., 2020). Epidemiological studies reveal that the incidence of injuries among non-professional table tennis players is impacted by several biomechanical risk factors (Teo et al., 2021). The existence of a causal relationship between postural asymmetry and rotator cuff injuries and lumbar spine pathologies is proven by systematic reviews (Ferrandez et al., 2021). Notable differences in landing patterns were seen for the adolescent group when compared to adult athletes (Avedesian et al., 2020), and the changes within the postural parameters in the sagittal posture added to the complexity of the assessment process (Ludwig et al., 2025). The physical attributes and postural changes presented by young athletes reflect the varied patterns of development (Santos et al., 2025), and the asymmetry assessments among 12-year-old soccer players reveal the critical point of development (Stefansdottir et al., 2025). Biomechanical analysis shows that players of table tennis and tennis have systematic changes of posture during prolonged training (Hitansha et al., 2025). The differences of biomechanical parameters between the forehand and backhand strokes of elite players provide a fundamental basis for injury analysis (Zhang, 2025). Bilateral differences of running gait parameters have speed-dependent discrepancies (Cartón-Llorente et al., 2024), and the relationship between posture and movement pattern quality has a direct predictive role in injury occurrence (Koźlenia and Kochan-Jacheć, 2024).

Traditional approaches to evaluating posture are mostly based on subjective observation and analysis of two-dimensional images, which makes them less capable of reflecting the three-dimensional dynamic qualities of table tennis. The dynamic parameters of forehand strokes in table tennis show considerable intra-individual differences (Bańkosz and Winiarski, 2020). According to a systematic review, there is no standardized methodology in current biomechanical studies (Wong et al., 2020). While elastic bands and other interventions have been shown to improve technical athletic movements among young athletes (Nikolakakis et al., 2020), actual quantitative monitoring of postural asymmetry is not possible. Though frameworks for the biomechanical quantification of visual markers have developed (Lyu et al., 2025), current applications were limited by the expense and complexity of the equipment.

In recent years, deep learning strategies have shown considerable potential in the prediction of sports injuries, and machine learning techniques have increasingly been incorporated into the process of risk assessment for injuries (Van Eetvelde et al., 2021). The application of machine learning in the prediction of sports injuries has shown that algorithmic techniques are more accurate than statistical ones (Amendolara et al., 2023). Sports injury diagnosis using artificial intelligence is a multi-dimensional methodological approach (Musat et al., 2024) to which a narrative literature review can integrate the experience with challenges of machine learning to predict injuries (Yuan et al., 2025). The scope-based survey identifies differences in algorithm performance using evidence synthesis (Leckey et al., 2025). Vision-based methods for human pose estimation can map data end-to-end with deep learning (Lan et al., 2022), and sports-related computer vision applications have progressed to deal with complicated injury analysis (Naik et al., 2022). The model for predicting non-contact injuries for the lower limbs with high sensitivity has adopted a multimodal fusion approach with interpretable machine learning (Huang et al., 2022). The combination of image coding with deep learning provides a new technical route (Ye et al., 2023). Deep models with a focus on multimodal data analysis prove their merits in prediction tasks (Zhang et al., 2023), and the use of deep learning in analyzing sporting performance exemplifies the importance of algorithm optimization (Jia et al., 2025). Systematic reviews are able to measure the rate at which performance advancements are being made by AI algorithms (Pietraszewski et al., 2025), whereas survey studies reveal that computer vision methods are revolutionizing the sport sector. In addition to that, survey studies confirm that computer vision algorithms are revolutionizing sports (Rossi et al., 2025). The combination of computer vision and deep learning represents the whole technology stack (Müller-Budack et al., 2025).

While deep learning methods have demonstrated substantial progress in human pose analysis, recent pose estimation frameworks such as HRNet (Wang et al., 2020), ViTPose (Xu et al., 2022), and RTMPose (Jiang et al., 2023) achieve impressive keypoint localization accuracy on benchmark datasets yet predominantly target adult populations in controlled settings or generic action recognition tasks. Contemporary approaches to sports injury prediction similarly focus on professional adult athletes with limited consideration of developmental biomechanics unique to adolescent populations undergoing skeletal maturation. Existing multimodal fusion architectures for sports analysis emphasize performance optimization such as technique classification rather than injury risk assessment grounded in biomechanical asymmetry quantification. Current methodologies exhibit critical deficiencies regarding age-specific modeling that accounts for growth-related changes in joint laxity and muscle strength imbalances, insufficient adaptation to sport-specific biomechanical constraints particularly the unilateral force generation patterns characteristic of racquet sports, and reliance on single-modality inputs that fail to capture the multi-dimensional nature of biomechanical risk.

This research addresses these limitations by constructing a multimodal deep learning framework specifically designed for posture asymmetry recognition and injury risk assessment in adolescent table tennis athletes. The proposed model integrates video RGB sequences, skeletal keypoint trajectories, and kinematic parameters through sport-adapted architectural components including weighted graph convolutions prioritizing unilateral kinetic chains and temporally-extended receptive fields matching stroke cycle durations, calibrated for the physiological constraints of developing athletes. The framework operates within a prospective screening paradigm wherein biomechanical asymmetry assessment serves as the initial stage in a multi-tiered injury prevention pipeline, analogous to established clinical screening instruments such as the Functional Movement Screen and Y-Balance Test that stratify injury risk through movement quality evaluation. This approach provides quantitative biomechanical profiling capabilities supporting data-driven intervention strategies, enabling early identification of athletes with elevated biomechanical loading patterns requiring confirmatory assessment and targeted preventive intervention before tissue damage manifests clinically.

## 2 Methodology

### 2.1 Problem definition and overall framework

**Figure 1:**
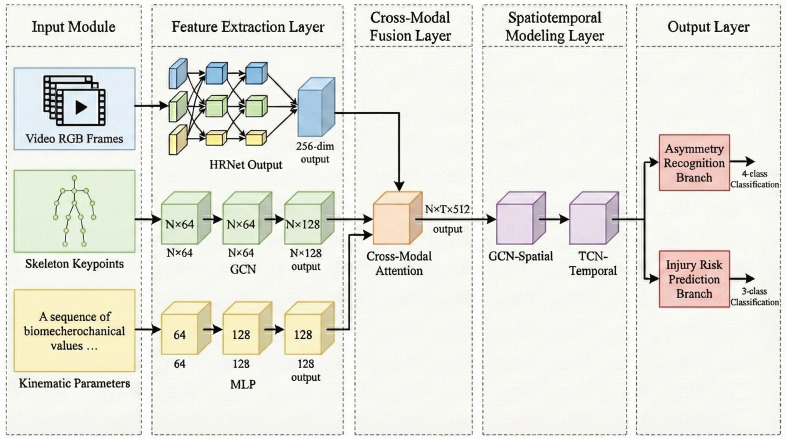
Shows the whole process of information processing from video input to risk assessment. In particular, the input layer processes stroke videos from TTStroke-21 and provides three modality data. The feature extraction layer extracts features through HRNet, GCN, and MLP. A cross-modal attention mechanism is used for adaptive weight learning. Spatiotemporal modeling is adopted to capture actions’ temporal patterns. Meanwhile, a dual-branch output layer provides recognition and predict results.

This study proposes a technical framework to recognize the asymmetry of posture and predict the sports injury risk in teen-aged table tennis athletes, framing the task as a classification problem in the multi-task learning approach. The posture asymmetry recognition task is defined as: given a table tennis stroke video sequence $V=\{v_{1},v_{2},\ldots ,v_{T}\}$, the model outputs an asymmetry type label $y_{asym}\in \{Normal,Shoulder\;Asymmetry,Trunk\;Asymmetry,Hip\;Asymmetry\}$; the injury risk prediction task estimates a biomechanical vulnerability level $y_{injury}\in \{Low\;Risk,Medium\;Risk,High\;Risk\}$ based on the extracted posture feature vector F. The term “injury risk prediction” refers to prospective biomechanical risk stratification for screening purposes rather than actuarial forecasting of injury occurrence, representing a clinically-grounded approach wherein expert sports medicine assessment translates measurable postural asymmetry patterns into actionable vulnerability categories. Low-risk indicates postural parameters within age-appropriate normative bilateral symmetry ranges; medium-risk reflects isolated threshold exceedances suggesting localized biomechanical stress warranting preventive attention; high-risk denotes compound multi-segment asymmetries indicative of kinetic chain disruption patterns associated with elevated injury susceptibility in adolescent overhead athletes based on clinical sports medicine experience. Given that athletes at the adolescent stage have incompletely ossified bone structures, uneven development of muscular strength, and sport-specific parameters due to the unilateral force generation pattern of table tennis, the design of the proposed model includes representation requirements along the lines of the three parameters mentioned. The full technical process consists of a cascaded framework including multimodal feature extraction, spatiotemporal relationship modeling, feature fusion, and parallel output in two tasks, which makes it possible to achieve automatic risk assessment from raw video data in an end-to-end way. In order to better describe the data flow paths of multimodal data between processing layers and hierarchical interactions between functional modules, a system-level architecture including an input layer, a feature extraction layer, a fusion layer, and an output layer is proposed, as shown in **Figure 1**.

### 2.2 Multimodal deep learning model architecture

The architecture consists of a data fusion model for processing three different data streams, namely the RGB video stream, keypoint stream, and kinematic parameter stream. The RGB video stream is used for extracting features with multiple scales based on the High-Resolution Network (HRNet), which is based on a parallel multi-resolution sub-network architecture and requires the fusion of semantic features from multiple resolutions achieved through the application of multi-scale fusion units. This is effective for the recognition of human pose features within unmanned aerial vehicle visions (Lee et al., 2022). Skeletal keypoint sequences contain 2D coordinate temporal data of 17 keypoints, which describe the topological position of the human skeletal structure using a Graph Convolutional Network (GCN). The GCN specifically focuses on the unique property of unilateral force production in table tennis to promote the lateral edge weights among the arm holding the racket, the torso, and the opposite-arm holding the racket, as well as the longitudinal edge weights among the shoulder joint, the torso, and the hip joint. Through three-dimensional posture analysis based on deep learning, a valuable approach to modeling the pertinent spatial information for human activity type identification is attained (Çalışkan, 2023). The extraction of three-dimensional biomechanical parameters from two-dimensional keypoint coordinates employs established depth estimation approaches integrating geometric constraints and anthropometric proportionality relationships. HRNet outputs 17 keypoint locations $\left(x_{i},y_{i}\right)$ in image coordinates, from which three-dimensional joint positions are inferred through perspective geometry combined with temporal consistency constraints across video frames. Depth information for each keypoint is estimated based on relative body segment proportions, leveraging prior knowledge of adolescent anatomical ratios wherein torso height, limb lengths, and joint spacing follow predictable developmental patterns for the 12–17 age range. Angular measurements such as shoulder rotation are computed from the reconstructed spatial coordinates using vector projections between adjacent joint segments, defining rotation planes through three-point geometric constructions at the shoulder girdle. Spinal curvature parameters are extracted via piecewise linear approximation of the cervical-thoracic-lumbar keypoint sequence in three-dimensional space, with lateral deviation angles quantified relative to the vertical reference axis established by the hip-shoulder midline. This reconstruction approach enables estimation of clinically relevant asymmetry indicators including bilateral shoulder rotation differences, spinal lateral flexion angles, and pelvic tilt measurements that serve as inputs to the subsequent biomechanical feature vector. The use of sequences of kinematic parameters helps in calculating the twelve biomechanical features, incorporating keypoint coordinates, which are then transformed into a 128-dimensional feature vector using the multilayer perceptron. Depth-aware pose estimation technology is applied to help in the precise calculation of a sequence of kinematic parameters in gait analysis for an exoskeleton (Wang et al., 2023).

**Figure 2:**
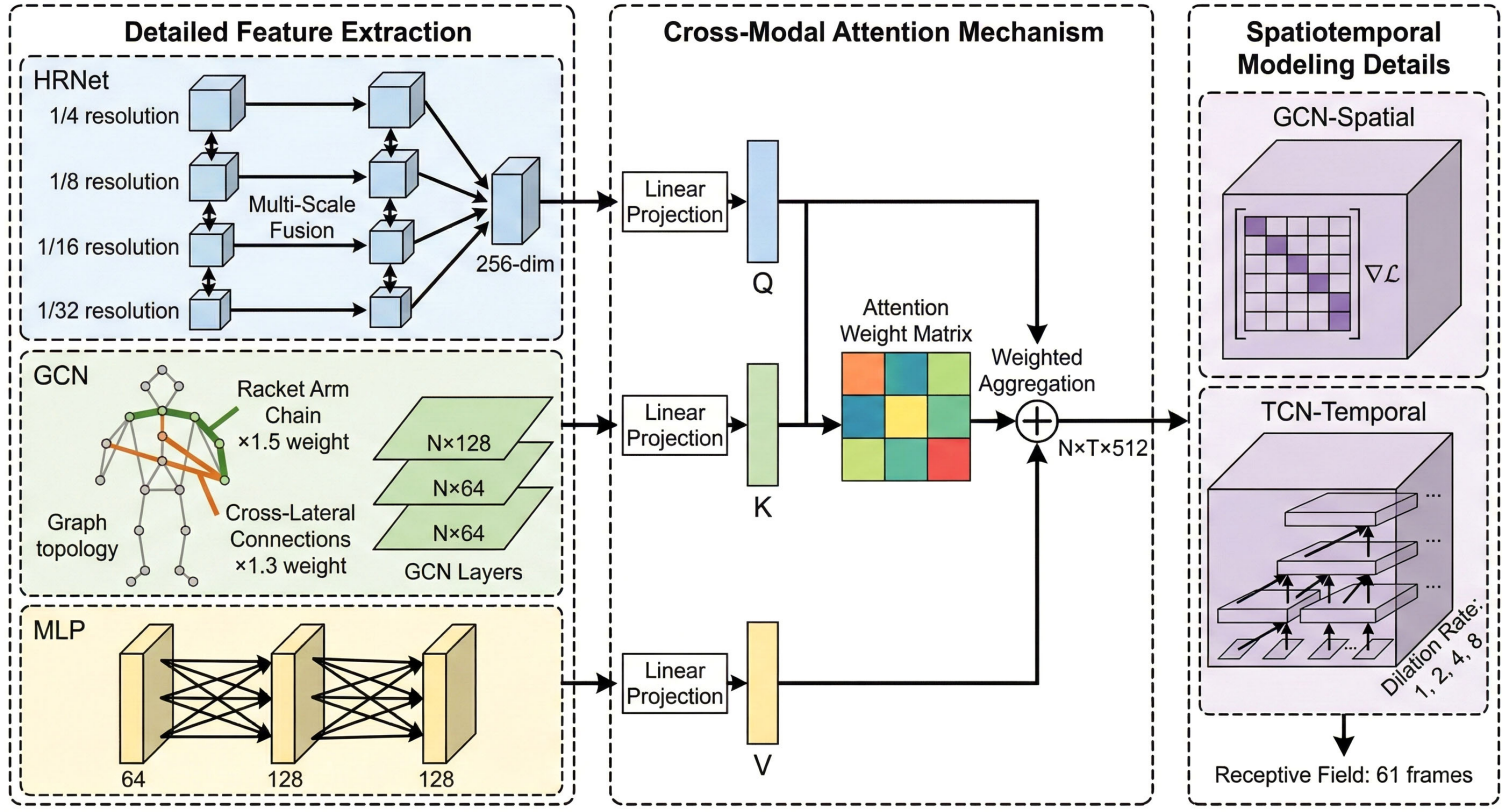
Presents the hierarchical structure of the model, where HRNet contains 4 parallel branches outputting 256-dimensional features, GCN adopts a 3-layer stacking structure and assigns 1.5x and 1.3x weights to the racket-holding arm chain and cross-lateral connections, the kinematic parameter encoder is a 3-layer MLP (dimensions 64-128-128), the cross-modal attention module calculates the correlations of the modalities to weigh the fusion, and the fused features of dimension 512 are put into the spatiotemporal modeling layer, which consists of GCN and TCN.

The spatiotemporal relationship modeling module adopts a serial structure of GCN and Temporal Convolutional Network (TCN), where the spatial dimension captures topological dependencies among joint points through GCN, with node feature aggregation following Equation 1:

(1) $$H^{\left(l+1\right)}=\sigma ({\tilde{D}}^{-\frac{1}{2}}\tilde{A}{\tilde{D}}^{-\frac{1}{2}}H^{\left(l\right)}W^{\left(l\right)})$$

where $\tilde{A}=A+I$ is the adjacency matrix with added self-connections, $\tilde{D}$ is the degree matrix, $H^{\left(l\right)}$ is the feature representation at layer l, and $W^{\left(l\right)}$ is the learnable weight matrix. The temporal aspect captures the dynamic process of stroke movements with TCN, which uses a causal dilated convolution with exponential growth rates as $2^{i}$. The design enables a 4-layer TCN to achieve a receptive field of 61, which captures the complete stroke process of the table tennis game. Because there are dramatic differences in semantic space and the size of features among the three modality datasets, this study proposes a cross-modal attention mechanism to achieve adaptive fusion of the contributions by learning the inter-modal correlations. In order to explain the concrete implementation of the feature extraction of modality, spatiotemporal modeling, and cross-modal fusion, a hierarchical network structure is designed in the research. The parameters and links of various modules are shown in **Figure 2**.

The cross-modal attention mechanism maps each modality feature to a unified query space and computes the attention weight matrix as shown in Equation 2.

(2) $$A_{ij}=\text{softmax}(\frac{Q_{i}K_{j}^{T}}{\sqrt{d_{k}}})\text{.}$$

obtaining the fused representation through weighted aggregation based on the weights $F_{fused}=\sum_{i=1}^{3}\sum_{j=1}^{3}A_{ij}V_{j}$, with this strategy dynamically capturing complementary information among modalities. The task output layer outputs a probability distribution for four classes in the posture asymmetry recognition branch, which uses a sequence of fully connected layers of sizes 256-128-64-4. For the injury risk prediction branch, it changes the output size to 3 to obtain a probability distribution for three classes. Both branches share features that facilitate multi-task learning to reuse features.

### 2.3 Table tennis-specific posture asymmetry quantification

The research focuses on the unilateral force creation characteristics of the table tennis and the physical attributes related to the growth and development phase of adolescence. It formulates an indicator system for the measurement of posture asymmetry in the sport, identifying the asymmetry characteristics pertaining to the shoulder, trunk, and hip regions of the body. Shoulder asymmetry indicators include the left-right shoulder joint rotation angle difference as shown in Equation 3.

(3) $$\Delta \theta_{shoulder}^{rot}=|\theta_{right}^{rot}-\theta_{left}^{rot}|$$

and the shoulder height difference as shown in Equation 4.

(4) $$\Delta h_{shoulder}=\frac{\left|y_{shoulder}^{right}-y_{shoulder}^{left}\right|}{H_{body}}$$

When $\Delta \theta_{shoulder}^{rot}>15{}^{\circ}$ or $\Delta h_{shoulder}>0.05$, shoulder asymmetry is identified. The threshold values were derived through integration of pediatric sports medicine evidence establishing normative bilateral symmetry ranges for adolescent overhead athletes. Shoulder rotation asymmetries exceeding 15-20° have been consistently associated with glenohumeral internal rotation deficit and scapular dyskinesis risk in youth racquet sport populations, with research demonstrating that horizontal adduction deficits greater than 15° in adolescent throwers may identify those at elevated injury risk. The 15° threshold aligns with established clinical cutoffs for pathological glenohumeral internal rotation deficit while accounting for adolescent-specific biomechanical characteristics including higher joint laxity and ongoing skeletal maturation in the 12–17 age range. Age-adjusted consideration of adult musculoskeletal disorder prevention thresholds (Piñero-Fuentes et al., 2021) provides additional supporting evidence, recognizing that identical asymmetry magnitudes carry different injury implications across developmental stages due to growth-related variations in joint stability and muscle strength ratios.

Trunk asymmetry indicators include spinal lateral flexion angle and trunk rotation angle, where the spinal lateral flexion angle $\alpha_{spine}$ is calculated through the three-dimensional spatial positional relationships of cervical, thoracic, and lumbar vertebrae points, and based on a quadratic curve $y=ax^{2}+bx+c$ fitted through three points, the lateral flexion angle is given in Equation 5:

(5) $$\alpha_{spine}=\arctan(2a\cdot x_{vertex})$$

where $x_{vertex}=-b/(2a)$ is the horizontal coordinate of the parabola vertex. Hip asymmetry indicators include hip joint flexion angle difference and pelvic tilt angle as shown in Equation 6.

(6) $$\gamma_{pelvis}=\arctan(\frac{h_{hip}^{right}-h_{hip}^{left}}{d_{hip}})$$

where $d_{hip}$ is the Euclidean distance between the left and right hip joint points, with asymmetry in anterior or posterior tilt potentially leading to uneven lumbar pressure, which has the potential to increase the risk of intervertebral disc damage.

The specific hip asymmetry thresholds ( Δθ>10° for hip flexion difference, γ>5° for pelvic obliquity) were established through adolescent-specific validation processes analogous to the shoulder threshold derivation. Lateral pelvic tilt exceeding 5° represents a clinically established threshold for pelvic obliquity in adolescent populations, with multiple studies documenting that sacral takeoff angles greater than 5° in adolescent idiopathic scoliosis patients associate with compensatory lumbar curve development and coronal imbalance. The 5° pelvic obliquity threshold has been documented as a precursor indicator for compensatory spinal adaptations in developing athletes, particularly under repetitive unilateral loading conditions characteristic of table tennis stroke mechanics where asymmetric force generation patterns may initiate progressive postural adjustments. The 10° hip flexion difference threshold aligns with normative asymmetry ranges observed in adolescent gait analysis, adjusted for the sport-specific demands of dynamic rotation and weight transfer patterns inherent to table tennis footwork. The precise quantification of these biomechanical parameters benefits from validated sensor fusion methodologies, as demonstrated by table tennis forehand stroke evaluation systems employing inertial measurement unit sensors combined with deep learning algorithms for accurate kinematic feature extraction (Tabrizi et al., 2021).

**Figure 3:**
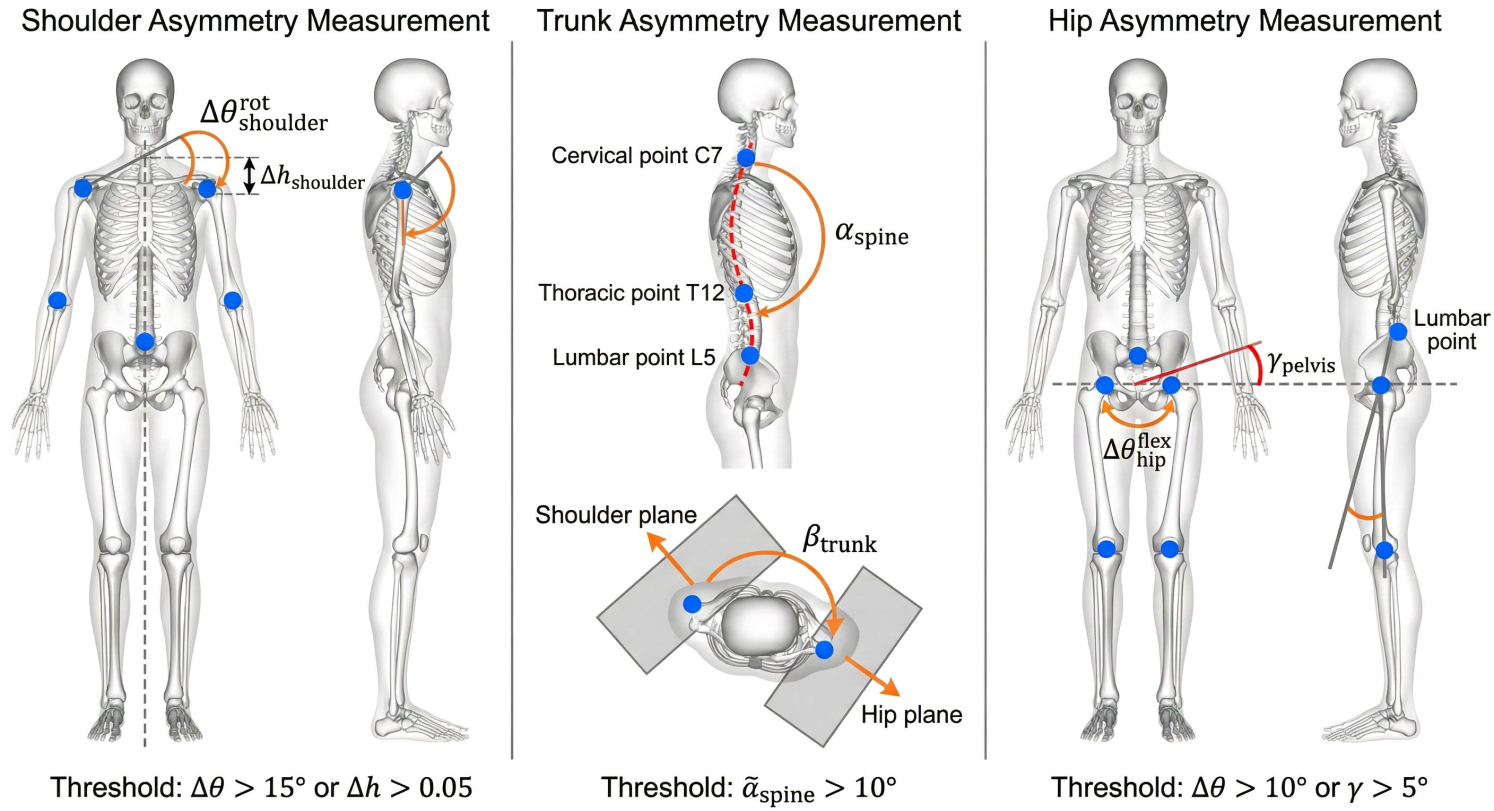
Shows the geometric evaluation of asymmetry indexes based on a three-dimensional skeletal model. The left side shows evaluation of differences in angles of shoulder joint rotation and shoulder height, while the middle part shows evaluation of angles of lateral bending of spine and trunk rotation. The right side shows evaluation of differences in angles of hip joint flexion and pelvic tilt.

Based on the diversity of the required postures for different stroke actions, the quantification approach develops assessment standards for different sport-specific actions, including forehand attack, backhand push, serve, and chop. When evaluating forehand attack actions, the range for the shoulder rotation angle difference is determined to be between 10° and 20°. Backhand push action assessment concentrates on differences in shoulder height and bilateral symmetry for the hips. Serve action assessment emphasizes the dynamic characteristics of lateral flexion and trunk rotation of the spine. To explain the geometric definitions regarding the multiple indicators of asymmetry and the spatial measuring strategies used on the human skeleton model, this research proposes a visualization computation framework based on a three-dimensional coordinate system. The computation strategies for measuring asymmetry indicators for each body part are shown in **Figure 3**.

The incorporation of considerations that are unique to adolescents involves verbatim mention of key physiological attributes such as high skeletal plasticity as well as high joint mobility that characterize this phase. The procedure involves a reduction in judgment criteria by 10-15% compared to adult participants while incorporating covariates such as height, weight, years of training, and biological age as shown in Equation 7:

(7) $$\theta_{norm}=\frac{\theta_{raw}-\mu_{age}}{\sigma_{age}}$$

where $\mu_{age}$ and $\sigma_{age}$ are the mean and standard deviation of athletes in the same age group respectively, ensuring comparability of evaluation results across different developmental stages.

### 2.4 Loss function design

The loss function is defined under a multi-task learning framework that combines the optimization tasks of posture asymmetry recognition and injury risk prediction. In posture asymmetry recognition, a cross-entropy loss function for the four-class problem is defined in Equation 8:

(8) $$L_{asym}=-\frac{1}{N}\sum_{n=1}^{N}\sum_{c=1}^{4}y_{n,c}\log({\hat{y}}_{n,c})$$

Injury risk prediction uses Focal Loss to balance the classes. With the relatively small number of high-risk samples compared to low-risk samples in training data (approximately 1:15), Focal Loss dynamically adjusts loss weights by introducing a modulating factor as defined in Equation 9:

(9) $$L_{injury}=-\frac{1}{N}\sum_{n=1}^{N}\sum_{c=1}^{3}\alpha_{c}{\left(1-\hat{y}n,c\right)}^{\gamma }y_{n,c}\log({\hat{y}}_{n,c})$$

where γ is the focusing parameter (set to 2), $\alpha_{c}$ is the class balance weight (3.0, 1.5, 1.0 for high/medium/low risk respectively), with this strategy demonstrating the ability to significantly improve model recognition accuracy for minority classes in multi-view sitting posture recognition (Cao et al., 2025).

The multi-task joint loss balances the optimization objectives of two sub-tasks through a dynamic weighting mechanism as shown in Equation 10:

(10) $$L_{total}=w_{1}(t)L_{asym}+w_{2}(t)L_{injury}+\lambda ||\Theta |{|}_{2}^{2}$$

where $w_{1}(t)$ and $w_{2}(t)$ are task weights dynamically adjusted with training epoch, and λ is the L2 regularization strength (1e-4). Dynamic weight adjustment is based on the uncertainty weighting method, adaptively balancing task importance by learning noise parameters $\sigma_{1}$ and $\sigma_{2}$ for each task as shown in Equation 11.

(11) $$w_{i}(t)=\frac{1}{2\sigma_{i}^{2}(t)}$$

A regularization component $\sum_{i=1}^{2}\log(\sigma_{i}(t))$ is added to the total loss to ensure that the noise variables do not tend to positive infinity, thus guaranteeing a converged training rate for all the tasks. This forms the technical reference for weight balancing techniques used within the robot vision-assisted human pose analysis technique for multi-task learning (Liu and Wang, 2025). The training process introduces regularization strategies including Dropout (ratio 0.5), Batch Normalization, and Early Stopping (terminating when validation loss does not decrease for 10 consecutive epochs), with data augmentation including random cropping (ratio 0.8-1.0), horizontal flipping (50% probability), temporal jittering (± 5 frames), Gaussian noise (standard deviation 0.01), and color jittering (brightness ±0.2, contrast ±0.2), with high-precision deep learning frameworks in sports action recognition demonstrating that reasonable regularization strategies can significantly reduce overfitting risk and improve model stability (Yang et al., 2025).

For ensuring the ability to replicate the experimental process and providing clear implementation details for other researchers, the entire process of the training procedure within the study is formalized using an algorithmic description. This includes key parts such as multimodal feature extraction, attention fusion for cross-modal processes, spatiotemporal modeling, dual-branch prediction, and dynamic weight update using uncertainty, as discussed in **Algorithm 1**.

Algorithm 1

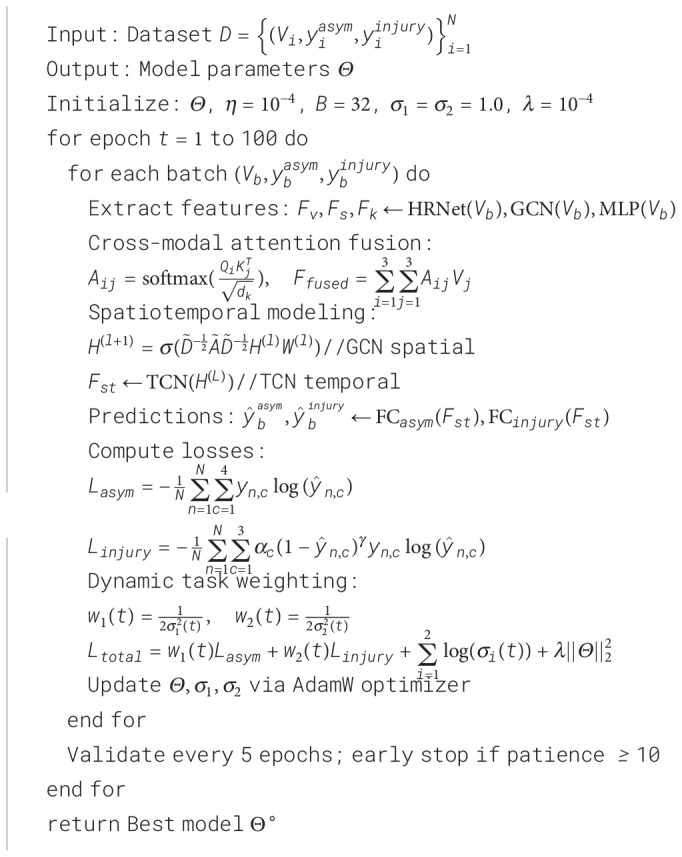

**Algorithm 1** illustrates the end-to-end training process for the multimodal deep learning architecture. The major breakthroughs include a cross-modal attention-based fusion approach and a task-weighting approach using uncertainty estimates. The training settings include a batch size of 32, the base learning rate of 1×10^−4^, a maximum of 100 training epochs, and early stopping strategy (patience=10), updating model parameters and noise parameters via AdamW optimizer.

## 3 Results

### 3.1 Dataset and experimental configuration

This study uses the TTStroke-21 dataset, which is available upon request from the MediaEval Sport Task organizers (https://multimediaeval.github.io/editions/2022/tasks/sportsvideo/). Access requires registration and acceptance of specific data use conditions as outlined by Martin et al. (2022) (Cao et al., 2025), which is specifically constructed for adolescent table tennis scenarios and contains high-resolution video sequences of 21 standard stroke types. The original dataset comprises stroke actions from 412 adolescent athletes aged 12 to 17 years captured during authentic training sessions, containing 30-frame RGB video sequences with skeletal tracking capabilities enabling keypoint extraction. The original dataset annotations focused on stroke type classification and temporal action localization for multimedia analysis applications. For biomechanical injury assessment purposes, the video sequences underwent expert re-annotation by three certified sports medicine physicians who independently evaluated each sample to assign pose asymmetry classifications (normal, shoulder asymmetry, trunk asymmetry, hip asymmetry) and injury risk levels (low risk, medium risk, high risk) based on quantitative asymmetry measurements and qualitative movement pattern assessment as detailed in the labeling protocol described subsequently. These biomechanical annotations constitute contributions beyond the original multimedia dataset scope, transforming the action recognition resource into a sports injury biomechanics research dataset. The data is split into training, validation, and testing sets with a ratio of 70%, 15%, and 15%, containing 8,624, 1,848, and 1,852 samples, respectively. Frame resolution is uniformly set to 512×512 pixels in the preprocessing phase, keypoint positions are scaled using Z-normalization to cancel out the size difference, and kinematic variables are computed using a sliding window with a size of 5 frames. The annotation process was performed by three sports medicine professionals conducting independent assessments. Inter-rater reliability analysis yielded Fleiss’ Kappa κ = 0.84 (95% CI: 0.81-0.87) for posture asymmetry type classification (normal vs. shoulder vs. trunk vs. hip), and κ = 0.78 (95% CI: 0.74-0.82) for injury risk level assignment (low vs. medium vs. high). Both values exceed the 0.75 threshold for substantial agreement, confirming reliable consensus across expert evaluators. The slightly lower agreement for risk stratification reflects the inherently greater subjectivity in prognostic judgment compared to descriptive asymmetry categorization. The class distribution exhibits imbalanced characteristics, with pose asymmetry categories distributed as normal posture 38.2%, shoulder asymmetry 27.5%, trunk asymmetry 21.3%, and hip asymmetry 13.0%, and injury risk levels distributed as low risk 62.8%, moderate risk 30.5%, and high risk 6.7%.

The injury risk labels were assigned through a consensus protocol integrating quantitative measurements with clinical judgment. Three sports medicine physicians with specialized expertise in adolescent athletic injury biomechanics independently evaluated each video sample alongside extracted asymmetry parameters including shoulder rotation differences, spinal lateral flexion angles, pelvic tilt measurements, and bilateral height asymmetries. Low-risk classification required all indicators within established normative ranges for adolescent bilateral symmetry as documented in pediatric sports medicine literature. Medium-risk designation applied when isolated parameters exceeded clinical thresholds without multi-segment involvement, such as shoulder asymmetry surpassing threshold values in isolation. High-risk categorization required simultaneous threshold violations across multiple body segments coupled with observable compensatory movement patterns during stroke execution. Consensus discussions systematically resolved initial rating discrepancies through reference to validated biomechanical assessment protocols.

This expert-derived labeling approach reflects current methodological constraints in adolescent sports injury research, where prospective injury surveillance over extended follow-up periods remains logistically challenging and resource-intensive. The risk classifications represent expert clinical translation of biomechanical asymmetry evidence into vulnerability stratification rather than epidemiologically-validated injury probabilities, acknowledging that actual injury outcomes depend upon multifactorial interactions including training load, fatigue accumulation, technique progression, and individual pain tolerance thresholds not captured in postural assessment alone. Three fundamental aspects require explicit clarification to prevent misinterpretation of model capabilities. The model predicts biomechanical risk scores rather than actuarial injury probabilities, with risk categories representing expert-assessed postural deviation severity rather than epidemiologically-calibrated incidence forecasts wherein high-risk classifications indicate elevated biomechanical loading patterns warranting preventive attention rather than definitive injury prognosis. Expert annotation introduces inherent subjectivity despite consensus protocols, with potential biases including threshold interpretation variability across evaluators, differential weighting of compensatory movement patterns, and systematic tendencies toward conservative or aggressive risk classification influenced by clinical training backgrounds, though inter-rater reliability metrics achieving κ=0.84 for asymmetry and κ=0.78 for risk stratification provide quantitative evidence for annotation consistency. Labeling standard consistency was maintained through standardized asymmetry measurement procedures applied uniformly across all samples, consensus-driven resolution of rating discrepancies through systematic reference to validated biomechanical thresholds documented in Section 2.3, and longitudinal evaluator calibration sessions ensuring interpretation stability across the annotation period. Nevertheless, expert-assessed biomechanical risk stratification serves established clinical utility as a screening framework for identifying athletes requiring enhanced monitoring or preventive intervention, consistent with standard practice in sports medicine where intervention decisions often precede definitive injury occurrence. The model developed in this study should be understood as automating and standardizing the expert biomechanical assessment process to enable scalable screening implementation, rather than replacing prospective epidemiological injury prediction which would require longitudinal cohort designs beyond the current study scope.

The absence of prospective injury outcome data constitutes the fundamental methodological limitation requiring explicit acknowledgment. Model predictions identify postural deviation patterns that expert clinicians associate with elevated biomechanical loading, yet validation against actual injury incidence remains necessary to establish predictive validity. Prospective cohort investigations tracking baseline risk classifications through competitive seasons would enable calculation of sensitivity, specificity, and positive predictive value for injury occurrence, calibration of classification thresholds against observed injury rates, and identification of specific injury types linked to particular asymmetry patterns. Until such validation occurs, the risk categories should be interpreted as biomechanical stress exposure indicators informing targeted monitoring strategies rather than definitive injury forecasts. Clinical implementation should position the system as a screening tool triggering confirmatory biomechanical assessment by qualified practitioners rather than autonomous diagnostic decision-making.

To comprehensively evaluate the performance of the proposed method, six representative baseline methods are selected for comparison, including OpenPose+SVM, HRNet+LSTM, ST-GCN, ViTPose+Transformer, RTMPose+TCN, and Pose-C3D. All comparative methods are pretrained on the COCO dataset and then fine-tuned on the TTStroke-21 training set, with training configurations uniformly set to learning rate 0.0001, batch size 32, AdamW optimizer, and weight decay 0.0001. Evaluation metrics include PCK@50 for pose estimation, F1-score for pose asymmetry recognition, AUC for injury risk prediction, single-frame inference time, and model parameter count.

### 3.2 Pose estimation performance validation

**Figure 4:**
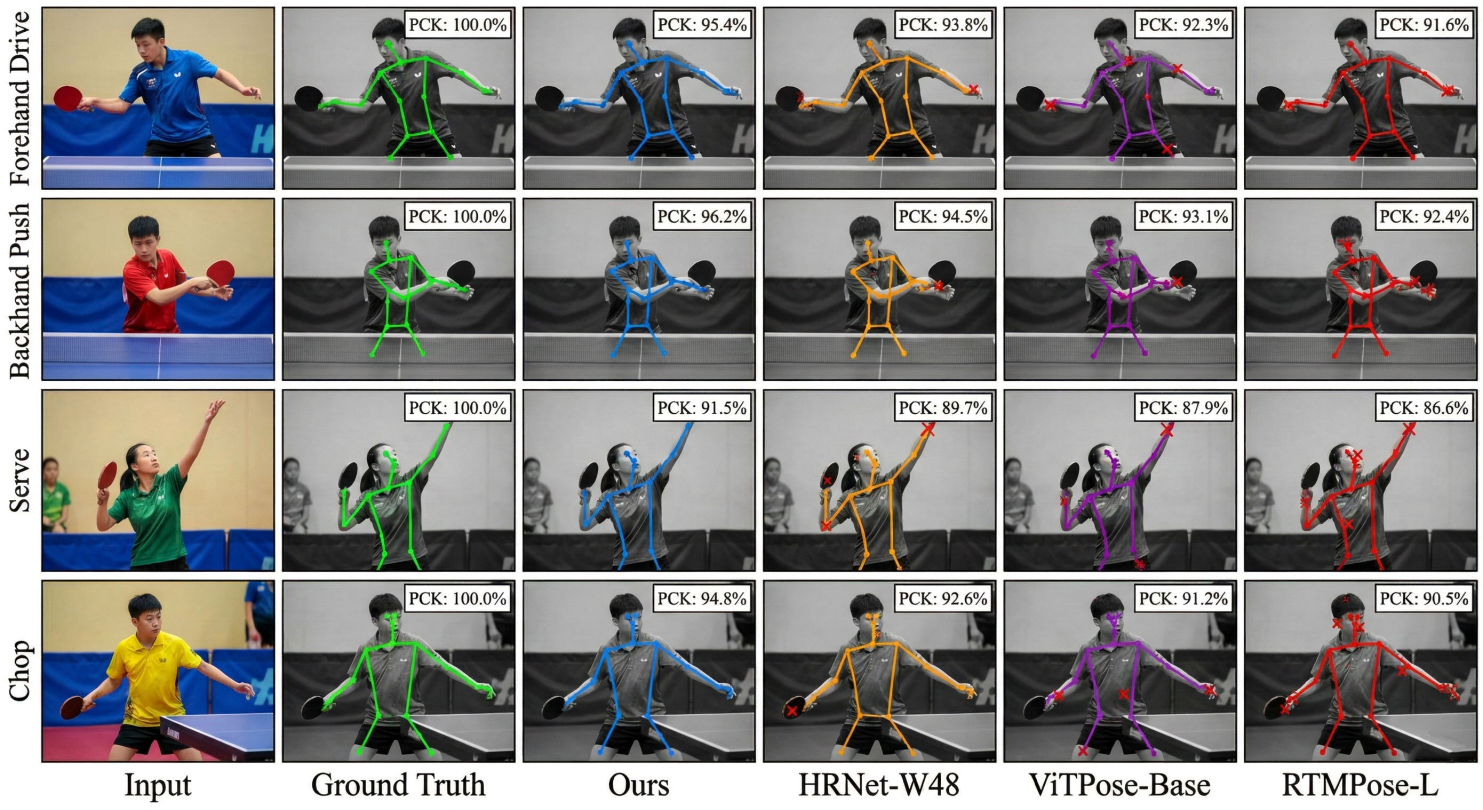
Higher detection accuracy is obtained using the proposed approach compared to the existing approaches for all four types of shots. The maximum accuracy is obtained for forehand attack and backhand push shots. The serve action is most difficult to recognize because of large body rotation and occlusion; however, the proposed approach can maintain quite accurate results in this action.

Pose estimation is the core component, in which the accuracy of keypoint detection has a direct impact on the task. To analyze the ability of the proposed method in table tennis-related scenarios, a series of experiments were carried out to compare the improved HRNet network with three prevailing methods: HRNet-W48, ViTPose-Base, and RTMPose-L. Assessment relies on the PCK50method, which considers torso diameter to be the distance from the left shoulder to the right hip. This is more relevant to adolescent sports players of different heights than specific pixel values. The work is focused on four typical strokes of table tennis: forehand drive, backhand push, serve, and chop, covering the basic categories and levels of complexity of table tennis strokes. Based on the **Figure 4**, the comparison on the accuracy of keypoint detection between different approaches for the four strokes proves the efficacy of the new approach.

### 3.3 Performance of pose asymmetry recognition

**Figure 5:**
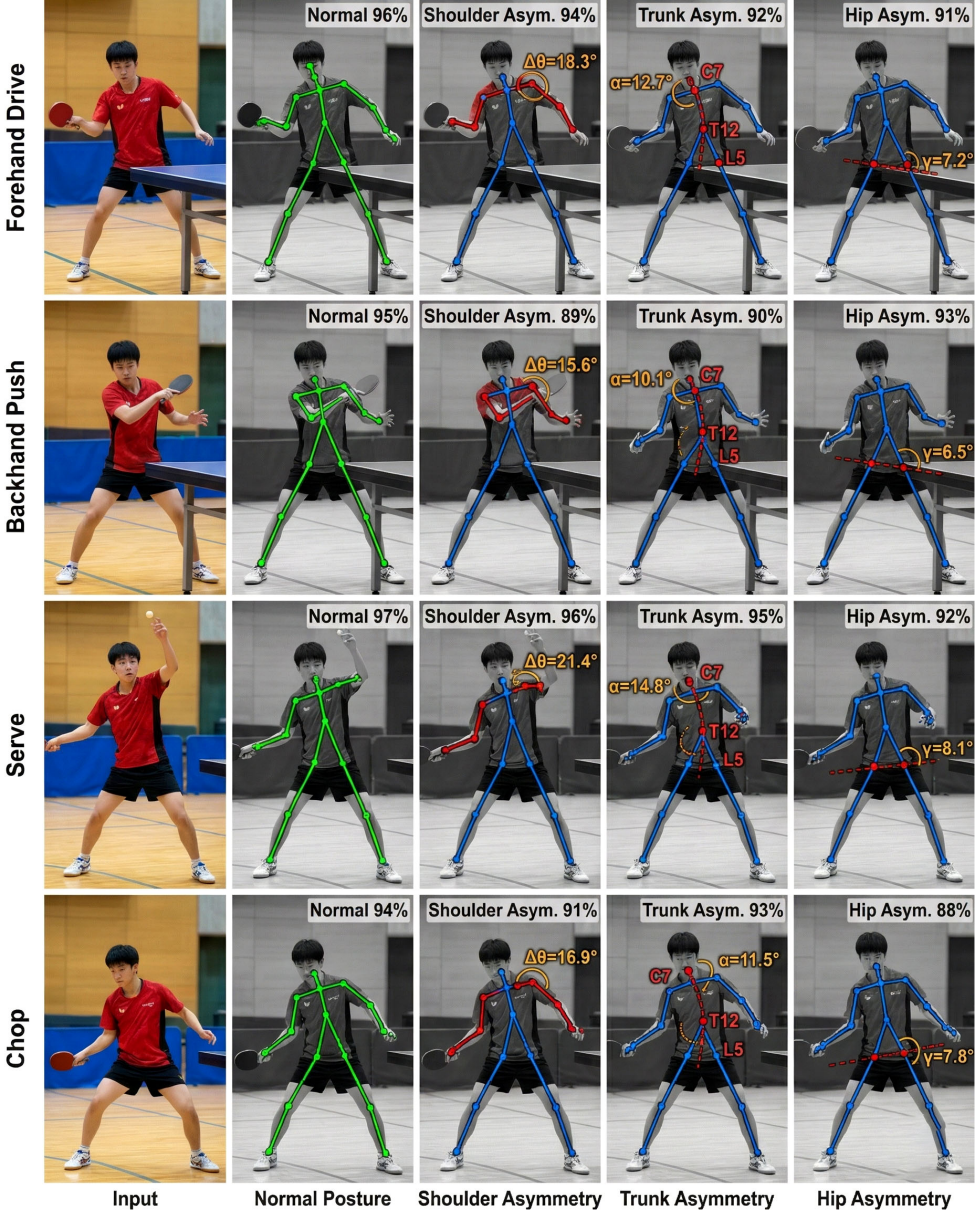
Illustrates that the model has captured well the variations in the characteristics of posture based on the type of stroke. Forehand attack has right shoulder external rotation of 18.3° more than left shoulder, serve action has spine lateral flexion of 14.8°, and chop action has tilt of pelvis of 7.8°, while the prediction confidence is over 88%.

The goal of posture asymmetry recognition is to automatically detect any deviation in posture patterns that could be apparent in adolescent table tennis players during stroke completion. This is hindered by class imbalance and decision boundary uncertainty due to the biomechanical relationship between trunk and hip asymmetry. In order to solve these challenges, a multi-modal feature fusion strategy is used in the model, and the video RGB sequence, skeleton keypoint sequence, and kinematic parameter sequence are fused adaptively through a cross-modal attention mechanism. The training algorithm uses the class-weighted cross-entropy loss function, where the class weight coefficient is set according to the proportion of each class, thus dealing with the issue of class imbalance. In order to intuitively explain the performance of the model on shot recognition, based on the four typical shot types, the sample skeletons for analysis are extracted. The asymmetric areas are code-colored, and the important angle indexes are measured. The skeleton detection results on strokes for five pose types are shown in **Figure 5**.

**Figure 6:**
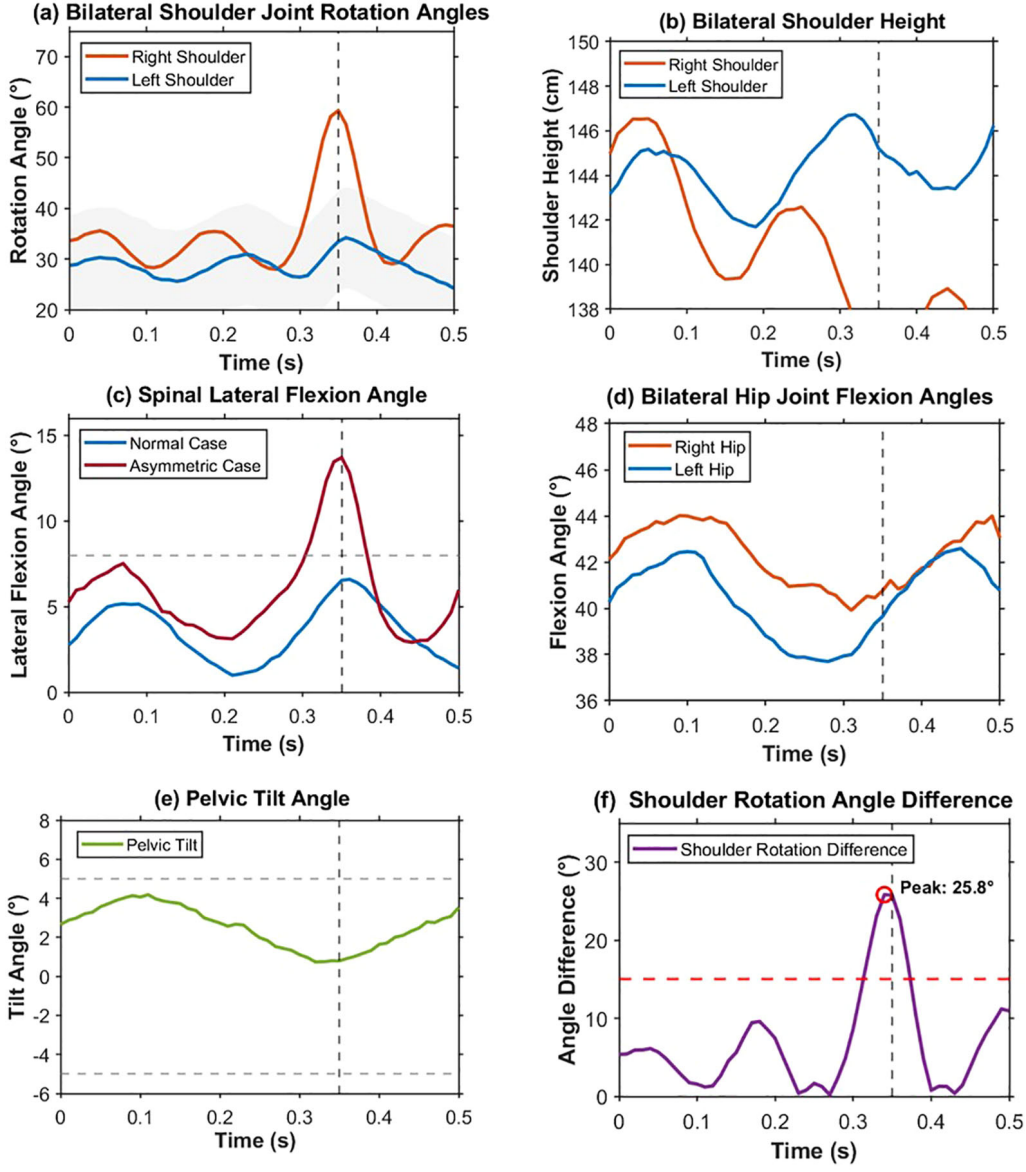
Illustrates the sequence of the six major indicators during the forehand attack, where the black vertical dashed line represents the contact point (t = 0.35s). **(a)** shows that the rotation Angle of the right shoulder joint reach their maximum during the instant of the stroke, and the left shoulder joint is relatively stable. **(b)** shows that the graph of the elevation of the bilateral shoulder has an inverted trend. **(c)** shows that the value of the lateral spinal flexion Angle in the asymmetric model greatly goes beyond the normal limit when the stroke occurs (gray dotted line 8°). **(d)** shows that the bilateral hip flexion Angle was quite stable. **(e)** illustrates that the difference in pelvic tilt Angle was always within the normal range (gray dashed line ±5°). **(f)** illustrates that the shoulder rotation Angle was 25.8° at stroke, well above the threshold (red dashed line 15°).

For the purpose of explaining the dynamic process of posture asymmetry evolution in the process of a hit, a case study of a forehand drive has been chosen. The forehand drive process duration takes about 0.3 to 0.5 seconds, and the case study uses a sliding window with a size of 5 frames and a step of 1 frame. Six major features are extracted: the rotation angles of the left and right shoulder joints; the vertical positions of the left and right shoulders; the lateral angle of spine flexion; the flexion angles of the left and right hip joints; the angle of pelvic incline; and the difference between the rotation angles of the shoulder joints. Such indicators represent the time course of asymmetry feature variations in shoulder, trunk, and hip values. As shown in **Figure 6**, the time courses of the six indicators over a full hitting motion show how posture asymmetry varies over time.

The performance differences appearing for various strokes and ages are related to the model learning to adapt to characteristics of ping pong performance and to the youth age group. Based on a sufficient sample and relatively standardized actions, the model had a maximum recognition rate for forehand attacks. The serving action is the most difficult to recognize, given the great complexity level involving a relatively small number of examples. The performance level for the 15 to 17-year-old adolescents is slightly higher compared to 12 to 14-year-old adolescents, due to more mature technical actions. The specific modeling approach for adolescents proposed within this study did counter the effects of individual differences during the period of growth development. The specific performance details for recognizing the pose asymmetry for various types of shots as well as various age categories can be seen within **Table 1**.

**Table 1:** Posture asymmetry recognition performance by stroke types and age groups.

Stroke type/age group	Sample size	Overall accuracy (%)	Normal F1 (%)	Shoulder asym. F1 (%)	Trunk asym. F1 (%)	Hip asym. F1 (%)	Trunk-hip confusion (%)
By Stroke Type							
Forehand Drive	602	85.7	90.2	85.1	82.3	83.8	5.8
Backhand Push	485	82.4	88.6	82.3	77.9	79.5	7.2
Serve	339	78.9	86.1	80.3	74.2	75.8	8.5
Chop	426	81.6	87.8	83.7	76.8	78.9	6.9
By Age Group							
12–14 years	915	79.8	87.0	81.2	77.5	79.1	7.5
15–17 years	937	82.5	89.5	84.1	81.2	80.8	6.1
Overall	1,852	81.2	88.3	82.7	79.4	80.1	6.8

**Table 1** shows that recognition accuracy in forehand attacks is higher than that in serves, and the difference in performance between the two age groups is limited to 3%. Shoulder asymmetry has shown to have a stable effect on recognition, and misclassification between torso asymmetry and hip asymmetry has an average value of 6.8%.

### 3.4 Damage risk prediction performance

**Figure 7:**
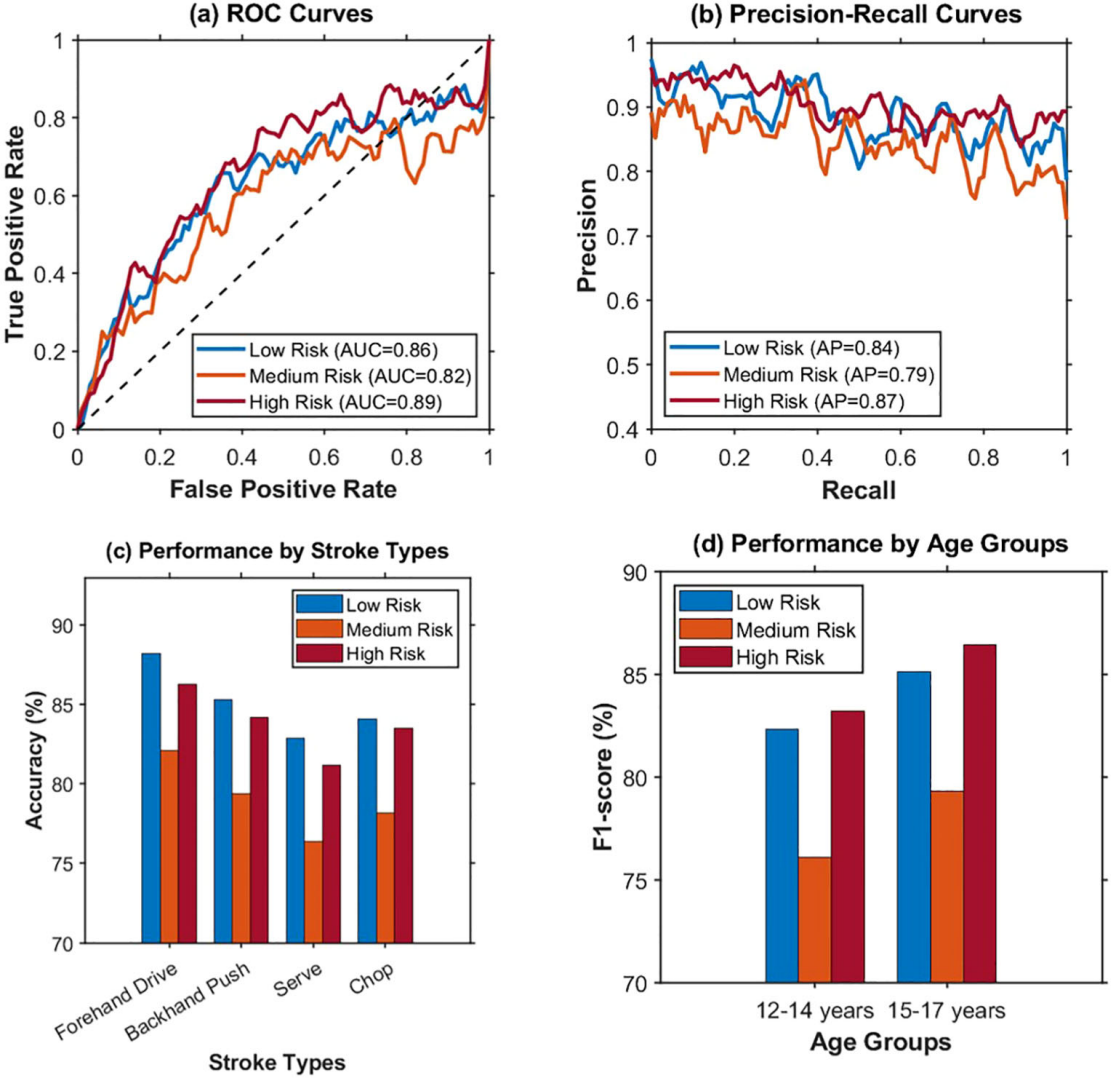
**(a)** Shows that the AUC of high risk is 0.89, and the AUC of medium risk is 0.82. **(b)** Shows that the AP of high risk is 0.87, which is higher than 0.79 of medium risk. **(c)** Shows that the prediction accuracy of forehand attack in the three risk levels is the best. **(d)** Shows that the 15–17 age group achieves higher F1-scores than the 12–14 age group across all three risk levels, with the largest gap observed in the High Risk category.

The aim of injury risk prediction task is to assess the injury-risk level of sports in adolescents of table tennis players, using posture asymmetry features as basis for evidence-based training and injury prevention. One of the main issues of injury risk prediction task is class imbalance, as 62.8% of data belongs to the low-risk class, 30.5% to medium-risk class, and only 6.7% to high-risk class. Such a distribution describes the current situation of low incidence of high-risk injuries in young athletes, making it difficult for the cross-entropy loss function to treat both classes fairly during training. To deal with this problem, Focal Loss is used as a goal for optimization in the damage risk prediction task. The weight for modulation is set at a value of 2.0. The weights for class coefficients are set at a value of 1.0 for samples with low risk, at 1.5 for samples with medium risk, and at 3.0 for samples with high risk. The multi-task joint training uses a dynamic weight update mechanism based on uncertainty. This enables the balance between posture asymmetry recognition tasks and injury risk prediction tasks. For a complete evaluation of the model’s prediction ability based on different levels of risk, ROC curves, Precision-Recall curves, as well as the differences in risk prediction ability based on different forms of batting and ages, are shown in **Figure 7**.

The differences in performance for different types of strokes and for different age groups are very significant in comprehending the adaptation of the model in the vertical domain. Analysis of the type of batting reveals that the performance difference mostly appears in the recognition accuracy of medium-risk samples. The properties of medium-risk samples are in between the properties of low-risk and high-risk samples, resulting in a high degree of ambiguity at the boundary. Age group analysis revealed a point of incongruity concerning the predictive validity for high-risk samples between the 12-14-year and 15-17-year age groups, possibly related to the stability of the postural control pattern. Feature importance analysis revealed the top three most important features to be the shoulder external rotation angle, the lateral flexion of the spine angle, and the pelvic tilt angle. This is consistent with the knowledge that exists in the field of sports medicine concerning the mechanisms of injury in the game of table tennis. The results are shown in **Table 2**.

**Table 2:** Injury risk prediction performance by stroke types and age groups.

Stroke type/age group	Low risk acc (%)	Medium risk acc (%)	High risk acc (%)	Low risk AUC	Medium AUC	High AUC	Average acc (%)
By Stroke Type							
Forehand Drive	88.2	82.1	86.3	0.88	0.84	0.91	85.5
Backhand Push	85.3	79.4	84.2	0.86	0.81	0.89	83.0
Serve	82.9	76.4	81.2	0.84	0.78	0.86	80.2
Chop	84.1	78.2	83.5	0.85	0.80	0.88	81.9
By Age Group							
12–14 years (n=915)	82.3	76.1	83.2	0.84	0.79	0.87	80.5
15–17 years (n=937)	85.1	79.3	86.4	0.87	0.83	0.90	83.6
Overall (n=1,852)	83.8	77.7	84.8	0.86	0.81	0.89	82.1

**Table 2** presents the performance of prediction for different shots and different age groups at the three risk levels. The difference in prediction accuracy for forehand attack shots and serve shots at medium risk was 5.7% whereas for low and high risks it was 5.3% and 5.1% respectively. The gap in high-risk predictions for 12- to 14-year-olds and 15- to 17-year-olds was 3.2% points. For all types of strokes and age groups, predictions for medium-risk strokes had lowest accuracy.

Beyond aggregate metrics, clinical implementation necessitates detailed evaluation of minority class performance, where misclassification of high-risk cases carries disproportionate clinical consequences. **Table 3** presents class-specific performance indicators for the three-tier risk stratification system.

**Table 3:** Class-specific performance metrics for risk stratification.

Risk level	Test samples (n)	Precision (%)	Recall (%)	F1-Score (%)
Low	1263	89.7	91.8	90.7
Medium	465	73.2	76.4	74.8
High	124	84.6	82.3	83.4

Metrics calculated on test set. High-risk sensitivity 82.3%; specificity for excluding high-risk 98.9%; positive predictive value 84.6%; negative predictive value 98.7%.

**Table 3** reveals differential predictive accuracy across risk stratification levels. The high-risk category achieves precision of 84.6% and recall of 82.3%, indicating that among athletes flagged as high-risk, approximately 85% exhibit severe biomechanical deviations requiring intervention, while the system identifies 82% of actual high-risk cases. The sensitivity of 82.3% combined with specificity of 98.9% for binary high-risk detection demonstrates effective discriminative ability despite substantial class imbalance. The modest false negative rate of 17.7% suggests that approximately one in six biomechanically vulnerable athletes may escape initial screening, though analysis indicates misclassified cases predominantly involve borderline threshold violations where measured asymmetries marginally fall below clinical cutoffs. The false positive rate below 1.1% minimizes over-screening burden, with high precision ensuring efficient allocation of confirmatory assessment resources.

Clinical acceptability of these characteristics depends upon institutional risk tolerance and intervention cost profiles. The negative predictive value of 98.7% provides confidence that athletes classified as low or medium risk lack severe postural deviations warranting immediate corrective action, supporting risk-stratified protocols concentrating intensive assessment on model-flagged high-risk individuals. In screening contexts where preventive interventions carry low morbidity, such as targeted strengthening programs or technique coaching, the observed sensitivity-specificity tradeoff appears favorable for deployment. The medium-risk category exhibits lower precision of 73.2%, reflecting inherent ambiguity of borderline cases straddling normal and pathological thresholds, consistent with clinical decision-making challenges in intermediate zones where expert judgment demonstrates greater inter-rater variability.

**Figure 8:**
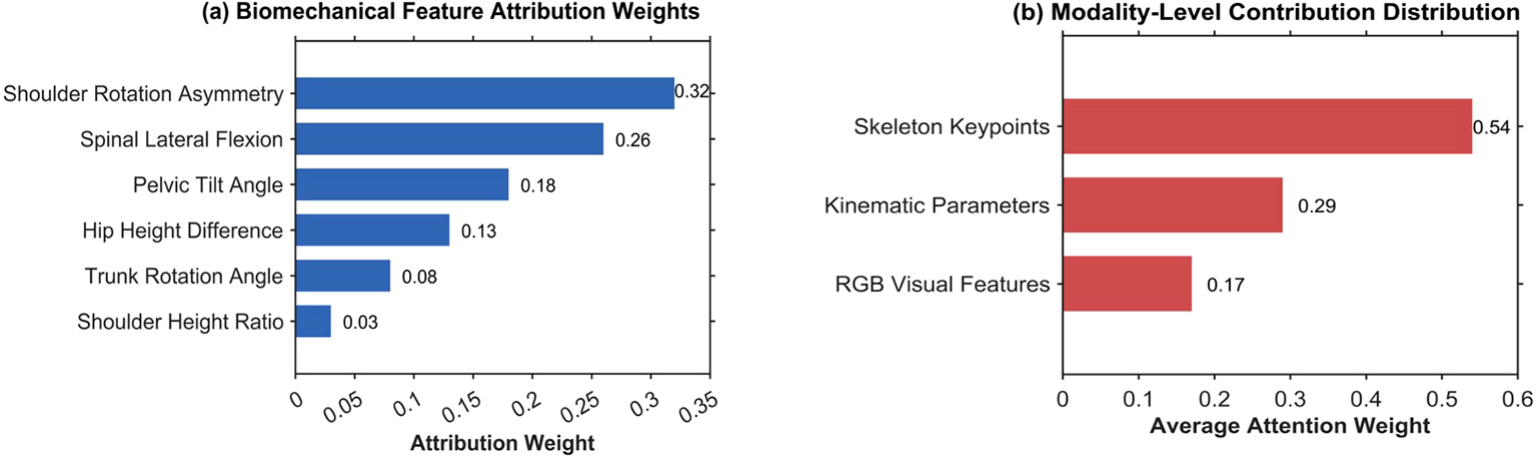
**(a)** Demonstrates that shoulder external rotation asymmetry constitutes the dominant predictive indicator with attribution weight of 0.32, followed by spinal lateral flexion at 0.26 and pelvic tilt angle at 0.18, collectively representing 76% of total attribution mass. These rankings align with established sports medicine knowledge wherein shoulder girdle imbalances represent primary musculoskeletal injury risk factors in racquet sports. Feature co-occurrence analysis reveals clinically plausible biomechanical mechanisms, with severe shoulder asymmetry exceeding 20°co-occurring with elevated spinal lateral flexion above 10° in 68% of high-risk instances, suggesting compensatory trunk tilting that disrupts kinetic chain integrity. Similarly, 61% of high-risk classifications exhibit concurrent pelvic tilt exceeding 6° alongside shoulder asymmetry, indicating force transmission pathway breakdown extending across lower and upper extremity segments. The model demonstrates threshold-based stratification logic consistent with clinical reasoning, classifying 76% of isolated single-parameter violations as medium-risk while compound multi-segment asymmetries involving two or more features trigger high-risk classification in 87% of cases, reflecting clinical understanding that multi-planar deviations impose greater systemic musculoskeletal stress than isolated abnormalities. **(b)** Reveals the multimodal integration mechanism underlying risk assessment, with skeleton keypoint features receiving the highest average attention weight of 0.54, reflecting their direct geometric representation of postural asymmetry, while kinematic parameter sequences contribute 0.29 through temporal dynamic information, and RGB visual features account for 0.17 primarily capturing global posture context. This modality weighting distribution indicates that the fusion architecture prioritizes explicit structural and motion cues over appearance-based patterns, aligning with clinical assessment practices that rely on measurable joint angles and movement parameters rather than visual inspection alone, thereby ensuring model predictions remain grounded in quantifiable biomechanical indicators amenable to practitioner verification.

Clinical adoption of deep learning diagnostic systems necessitates transparent decision pathways enabling practitioners to validate predictions through interpretable biomechanical reasoning rather than accepting models as opaque computational oracles. Integrated gradients analysis was conducted to quantify feature attribution patterns underlying injury risk assessments, revealing both the relative contribution of individual biomechanical parameters and the integration mechanisms across multimodal input streams. **Figure 8** presents feature importance rankings and modality-level contribution analysis for high-risk classifications.

### 3.5 Age-stratified performance analysis

The hypothesis that ages 12–14 constitute a critical intervention window requires empirical verification through age-disaggregated analysis. Although prior literature identifies this period as exhibiting heightened biomechanical vulnerability due to rapid skeletal growth, confirmation demands examining whether asymmetry severity and injury risk demonstrate measurable peaks within this range. The complete dataset was partitioned into single-year cohorts spanning ages 12–17 to detect nonlinear developmental trajectories obscured by binary grouping. The analysis evaluates three dimensions across age groups. Asymmetry indicator severity quantifies biomechanical imbalance through mean shoulder rotation differences and spinal lateral flexion angles. Injury risk distribution examines prevalence of high-risk classifications across developmental stages. Model prediction performance variability assesses whether classification difficulty varies systematically with age, potentially reflecting differential movement stereotypy across maturation phases. **Table 4** presents age-specific profiles synthesizing these analytical dimensions.

**Table 4:** Age-stratified asymmetry severity and model performance metrics.

Age (years)	N	Shoulder rotation Δθ (°) mean ± SD	Spinal flexion α (°) mean ± SD	High risk prevalence (%)	Model F1-asymmetry (%)	Model AUC-risk
12	63	11.7 ± 6.3	6.5 ± 3.4	7.9	76.2	0.827
13	68	14.3 ± 6.9	8.1 ± 3.8	8.8	78.5	0.851
14	72	16.1 ± 7.4	9.4 ± 4.3	11.1	80.9	0.879
15	71	14.5 ± 6.6	7.7 ± 3.6	7.0	83.4	0.888
16	69	12.8 ± 5.8	6.9 ± 3.1	6.1	84.7	0.896
17	69	12.1 ± 5.5	6.2 ± 2.9	5.1	85.1	0.905

Δθ represents bilateral shoulder rotation difference; α denotes spinal lateral flexion angle.

**Table 4** reveals nonlinear developmental trajectories for postural asymmetry. Shoulder rotation asymmetry peaks at age 14 with mean 16.1 ± 7.4°, representing 38% elevation from age 12 baseline, before declining through ages 15-17. Spinal flexion exhibits parallel patterns, peaking at 9.4 ± 4.3°for 14-year-olds. High-risk prevalence reaches maximum 11.1% at age 14, decreasing to 5.1% by age 17. Model performance shows inverse correlation with asymmetry severity, with ages 12–14 exhibiting lower F1-scores of 76.2-80.9% versus 83.4-85.1% for ages 15-17. One-way ANOVA confirms significant age effects with p<0.001, and *post-hoc* tests reveal age 14 differs significantly from both ages 12 and 16, substantiating a transient asymmetry amplification phase. While these cross-sectional comparisons provide empirical evidence supporting the 12–14 critical window hypothesis, longitudinal tracking of individual athletes across developmental stages would be required to establish definitive causal inferences regarding asymmetry progression trajectories.

### 3.6 Ablation experiment and comparative analysis

The ablation experiment is intended to extensively determine the effectiveness of each crucial module in the overall performance and to investigate the effect of removing or replacing a specific module on posture asymmetry identification and damage risk prediction. The experimental design covers six dimensions, which verify the effectiveness of multi-modal feature fusion strategy, cross-modal attention mechanism, GCN spatial modeling, TCN temporal modeling, pre-training strategy and loss function design, respectively. The cross-modal attention dimension is used to compare the performance gap of simple splicing and attention-based fusion. The GCN spatial modeling dimension validates the performance gain brought by the weighted edge aimed at capturing the unilateral force property in table tennis, compared with the GCN. The pre-training strategy dimension is used to determine the effect of pre-training on the COCO dataset in the process of transfer learning. The loss function dimension focuses on the effects of the cross-entropy loss, Focal Loss, and dynamic weights in handling the class imbalance. For all the ablation experiments, a similar setting has been used. These include a batch size of 32, learning rate of 0.0001, 100 training epochs, optimizer as AdamW, weight decay of 0.0001, and a Dropout probability of 0.5. As shown in **Table 5**, the performance comparison of the eight representative model configurations is averaged over three independent trainings.

**Table 5:** Ablation study results.

Model configuration	Posture asymmetry F1 (%)	Injury risk AUC (%)
Complete Model (Ours)	81.2	86.1
Single Modality		
- Video only	58.3	74.1
- Skeleton only	62.1	76.5
- Kinematic only	55.7	72.8
Multimodal Variants		
w/o Cross-modal Attention (Simple Concat)	76.1	82.0
w/o Weighted GCN (Standard GCN)	79.8	84.9
w/o 4-layer TCN (2-layer TCN)	79.5	84.6
w/o Pretraining	73.8	79.2
Loss Function Variants		
w/Cross-Entropy Loss	81.2	81.3
w/Focal Loss	81.2	84.2
Single-Task Baselines		
- Asymmetry Recognition Only	80.1	–
- Risk Prediction Only	–	84.8

As shown in **Table 5**, three-modal fusion brings an improvement of 19 percentage points, cross-modal attention brings an improvement of 5 percentage points, and pre-training and dynamic weights bring improvements of 7 and 2 percentage points, respectively. Single-task baseline comparison validates the multi-task learning framework’s feature-sharing benefits, with asymmetry-only training achieving F1-score of 80.1% and risk-only training achieving AUC of 84.8%, both inferior to the multi-task model’s 81.2% and 86.1% performance respectively. The 1.1 percentage point improvement in asymmetry recognition and 1.3 percentage point gain in risk prediction demonstrate mutual performance enhancement rather than negative transfer interference. Statistical correlation analysis between posture asymmetry classifications and injury risk levels reveals significant positive association with Pearson coefficient r=0.67 (p<0.001), indicating that samples exhibiting severe postural deviations systematically correspond to elevated risk categories, thereby supporting shared representation learning assumptions underlying the multi-task architecture. Task gradient direction analysis during training exhibits average cosine similarity of 0.73 between asymmetry and risk task gradients, confirming predominantly aligned optimization trajectories with minimal conflicting update signals that would suggest negative transfer effects.

The substantial performance gain from COCO pre-training merits examination of cross-domain transferability mechanisms despite apparent distributional mismatch between natural scene imagery and sport-specific stroke execution contexts. Although COCO depicts everyday activities with diverse environmental backgrounds while TTStroke-21 captures standardized athletic motions, several factors enable effective transfer learning including low-level visual feature universality wherein convolutional filters for edge detection and texture gradients generalize broadly across photographic domains, human body structural consistency through shared 17-keypoint skeletal topology despite kinematic differences between general actions and table tennis strokes, and pose estimation prior knowledge encoding spatial relationship constraints transferable across activity types. To assess domain proximity effects on transfer learning efficacy, ablation experiments compared alternative pre-training sources as presented in **Table 6**.

**Table 6:** Performance comparison of different pre-training strategies.

Pre-training source	Dataset characteristics	Posture asymmetry F1 (%)	Injury risk AUC (%)
None	Random initialization	73.8	79.2
COCO (adopted)	Natural scenes, 17-keypoint annotations	81.2	86.1
Human3.6M	Indoor motion capture, controlled poses	80.4	85.3
MPII	General human pose, diverse activities	79.1	84.7
Sports-1M	General sports videos, video-level labels	74.8	80.1

**Table 6** reveals that COCO pre-training achieves optimal performance despite depicting non-athletic scenarios, with Human3.6M yielding comparable results reflecting the value of precise pose annotations even in motion capture environments. MPII pre-training demonstrates moderate effectiveness, while Sports-1M provides minimal benefit likely due to coarse video-level annotation granularity insufficient for dense keypoint supervision. These comparisons indicate that pose estimation task alignment through fine-grained keypoint annotations matters more than visual domain similarity for effective transfer learning, validating the COCO pre-training strategy as a practical solution given limited availability of large-scale annotated sport-specific datasets.

Domain mismatch between general pose datasets and pediatric athletic populations introduces specific generalizability considerations requiring explicit acknowledgment. COCO and comparable pre-training sources predominantly feature adult subjects with mature skeletal proportions and movement kinematics, whereas the target application involves adolescent athletes aged 12–17 undergoing active skeletal growth characterized by distinct anthropometric ratios including higher limb-to-torso length variability, incomplete epiphyseal fusion affecting joint articulation patterns, and growth-related differences in movement coordination. These developmental characteristics potentially limit direct transferability of learned pose priors, as adult-derived feature representations may inadequately capture age-specific biomechanical constraints. Nevertheless, the empirical performance gains observed following COCO pre-training suggest that fundamental pose estimation capabilities including keypoint localization and spatial relationship modeling transfer effectively despite age-related distributional shifts, likely attributable to hierarchical feature learning wherein low-level edge and contour detectors generalize across age groups while subsequent fine-tuning on TTStroke-21 training data adapts higher-level representations to adolescent-specific morphological characteristics. Future investigations could quantify age-domain adaptation effects through comparative evaluation on purpose-collected pediatric pose datasets spanning broader activity contexts beyond table tennis, enabling assessment of whether adolescent-specific pre-training would yield incremental performance benefits justifying the substantial annotation costs required for dataset construction.

**Figure 9:**
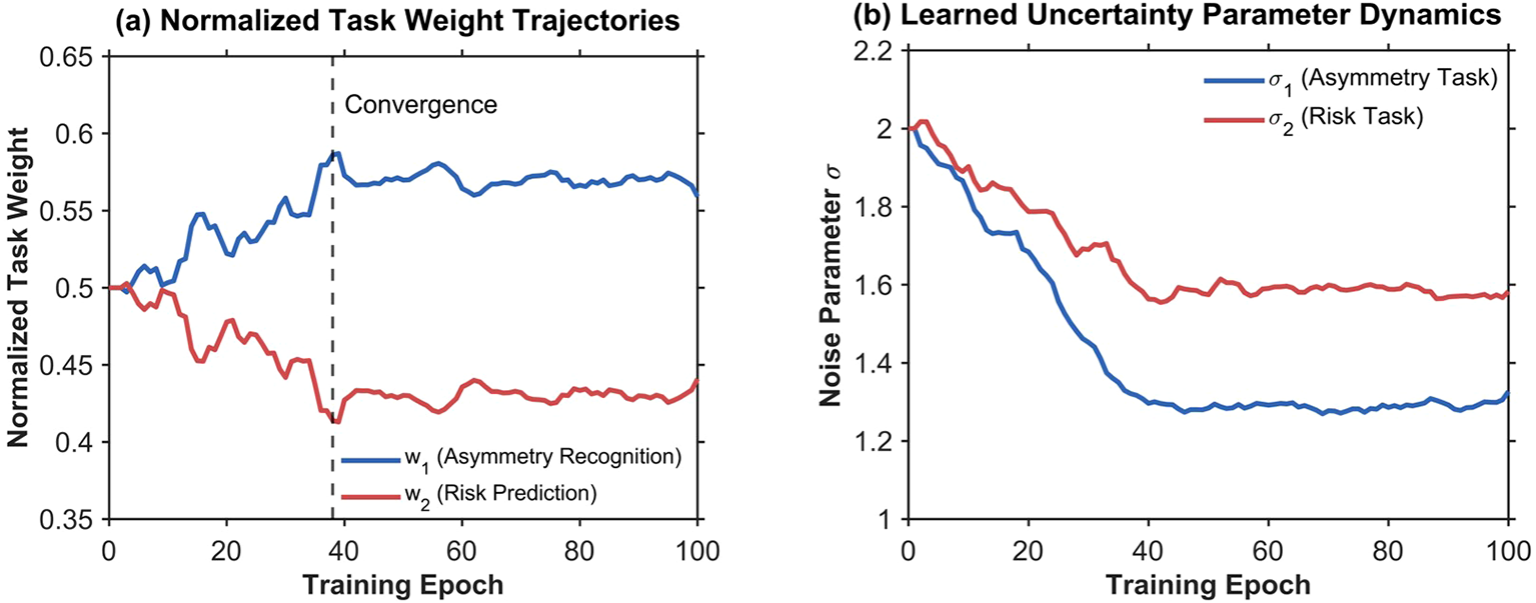
**(a)** Task weights stabilize around epoch 38, converging to w_1_ = 0.57 for asymmetry recognition and w_2_ = 0.43 for injury risk prediction, corresponding to learned uncertainty parameters σ_1_ = 1.29 and σ_2_ = 1.58 in **(b)**. The higher weight assigned to asymmetry recognition reflects its lower predictive uncertainty, indicating more confident predictions for four-class posture classification compared to three-level risk stratification. This weight distribution accords with task characteristics wherein postural asymmetry exhibits objectively measurable geometric features amenable to deterministic classification, whereas injury risk assessment involves greater inherent uncertainty due to individual biomechanical tolerance variability. The emergent 57:43 weight ratio suggests optimization naturally prioritizes robust asymmetry representations as foundational features for downstream risk inference, aligning with clinical intuition that structural postural deviations serve as observable precursors for biomechanical injury vulnerability. Ablation comparison with fixed equal weighting w_1_=w_2_ = 0.5 yields performance decrements of 1.7 percentage points in F1-score for asymmetry and 2.1 percentage points in AUC for risk prediction, validating the adaptive strategy’s contribution.

Beyond component-level ablation, the uncertainty-based dynamic weighting mechanism warrants examination regarding its adaptive optimization behavior. Unlike fixed weight schemes assigning predetermined task importance coefficients, the proposed approach jointly learns task-specific uncertainty parameters σ_1_ and σ_2_ that inversely modulate gradient contributions, enabling automatic balancing between asymmetry recognition and injury risk prediction based on relative predictive confidence. As training progresses, tasks exhibiting lower inherent uncertainty receive proportionally higher effective weights, reflecting learned epistemic assessment of each objective’s tractability. **Figure 9** illustrates the evolution of normalized task weights and uncertainty parameters across training epochs.

**Figure 10:**
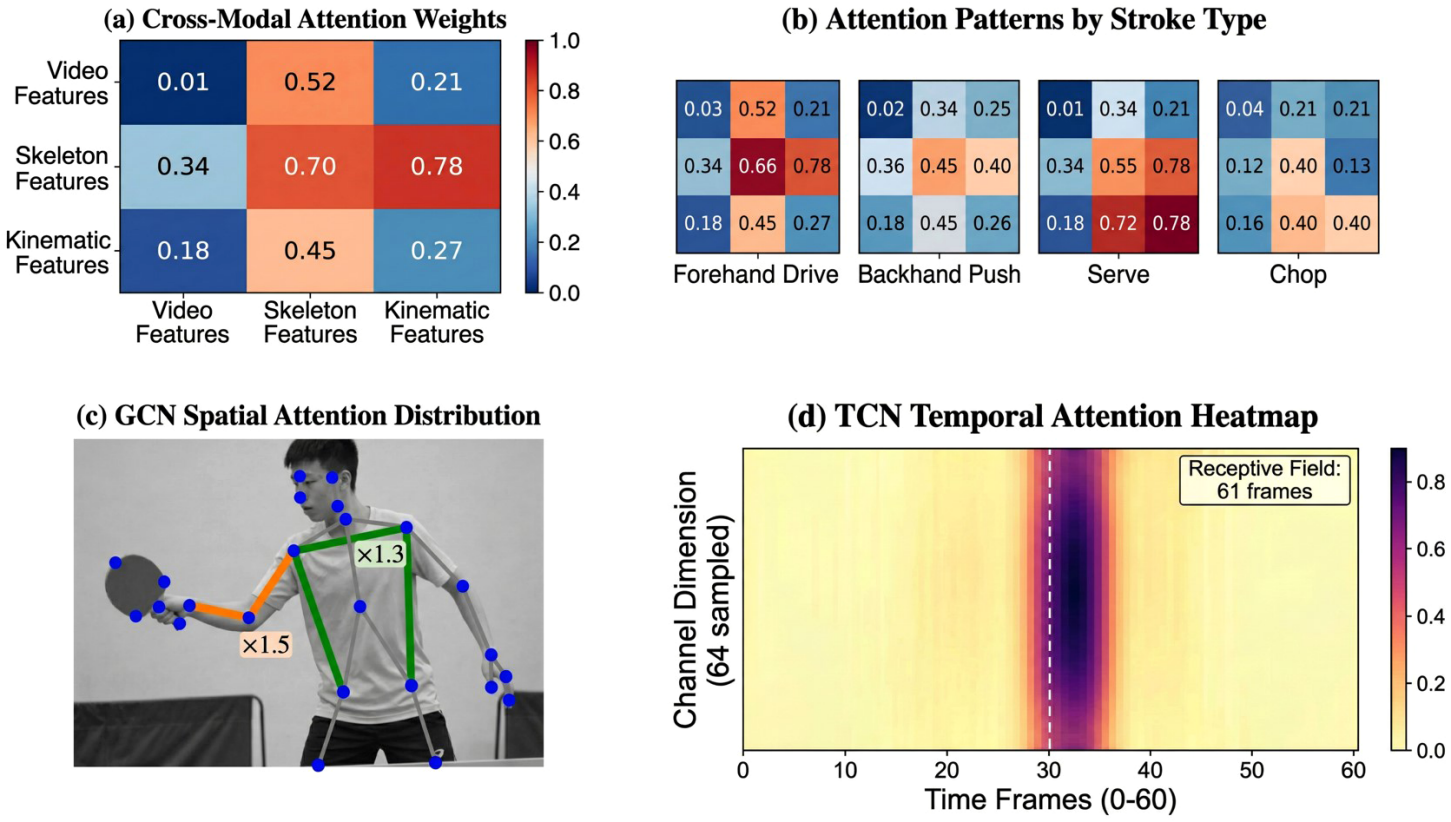
**(a)** Shows that skeleton features are highly dependent on kinematic features. **(b)** Shows that each stroke style has varying degrees of dependence on the modal. Forehand attack depends more on skeleton features while serve depends more on kinematic features. **(c)** Shows the GCN spatial attention distribution on the player’s skeleton, with the racket-arm chain edges weighted at ×1.5 and the cross-lateral connections weighted at ×1.3, reflecting the sport-specific asymmetric graph topology of table tennis. **(d)** Shows that the stroke instant corresponds to the highest temporal attention and the receptive field covers 61 frames.

For the sake of achieving a deep understanding of the operational principles of multi-modal fusion as well as the cross-modal attention mechanism, a visual analysis of the correlations between the modalities as well as the model’s reliance on the information sources is carried out. The weight matrix of cross-modal attention reflects the strength of interaction between video features, skeleton features, and kinematic features, and the larger values of the weight indicate a stronger role of the corresponding modality in the fused features. The analysis of attention patterns for different strokes aims to confirm the ability of the model to adaptively regulate reliance on different modalities in accordance with the type of task. For example, attack actions of a forehand may more significantly rely on the geometric information of the skeleton structure, whereas serve actions may more significantly rely on the information of acceleration in the kinematic data. The visualization of the GCN spatial attention shows the distribution of the weights of the edges of the graph corresponding to the characteristics of table tennis. The weight of the edge corresponding to the bat-holding bracelet is 1.5 times, and the weight of the edge corresponding to the cross-side connection is 1.3 times. The visualization of TCN temporal attention indicates the focus of the model on the temporal aspect, where the time of batting usually corresponds to the attention weight peak. **Figure 10** shows the visualization of the analysis of the picture over the four dimensions.

**Figure 11:**
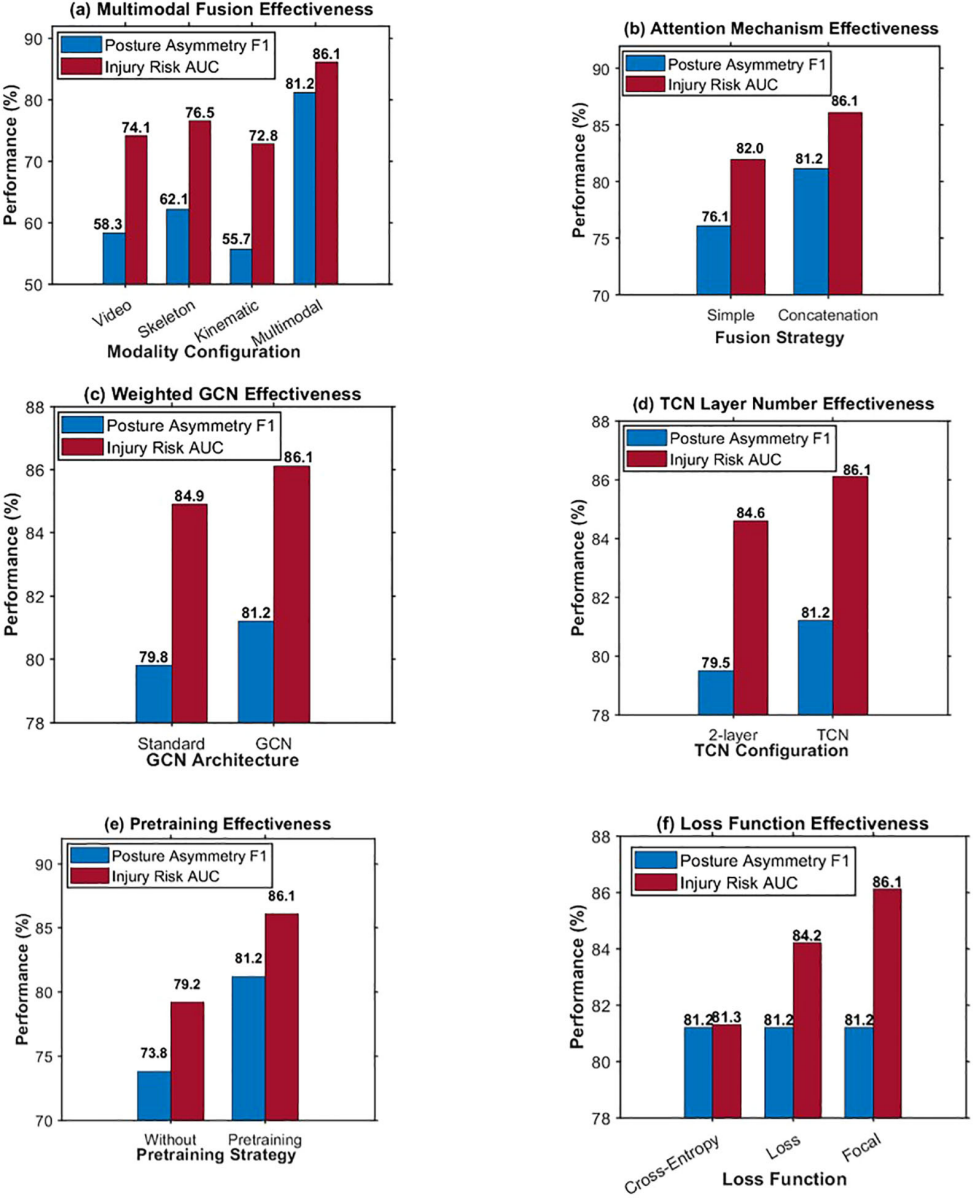
Shows the visualization results of ablation experiments in six dimensions, **(a)** shows that three-modal fusion performs much better than the single-modal configuration, **(b)** shows that cross-modal attention is better than simple concatenation, **(c)** shows that weighted GCN is better than standard GCN, **(d)** shows that 4-layer TCN is better than 2-layer TCN, **(e)** shows that pre-training brings significant performance improvement. **(f)** shows that the F1 of posture recognition remains consistent, whereas the AUC value of damage prediction presents an upward trend when employing the loss functions, and the optimal result is achieved by dynamic weighting.

In order to provide a visual representation of the ablation studies, a bar graph representing the results on six different dimensions has been shown in **Figure 11**. Since there are two colors in these columns, they illustrate the performance on both respective tasks: posture asymmetry identification and damage risk prediction.

To effectively evaluate the performance advantages of the proposed approach, it is compared with six baseline approaches using four performance metrics, including the F1-score for evaluating posture asymmetry recognition, the AUC for evaluating damage risk prediction, inference time per frame, and total model parameters. The inference times were tested on NVIDIA RTX 3090 with a batch size of 1 since it’s a real-time application, and their results were averaged over 1000 runs. The results for the seven methods are shown in **Table 7**.

**Table 7:** Performance comparison with baseline methods.

Method	Posture asymmetry F1 (%)	Injury risk AUC (%)	Inference time (ms)	Parameters (M)
OpenPose+SVM	68.1	74.3	45	25.6
HRNet+LSTM	73.5	78.2	58	41.2
ST-GCN	71.8	76.8	52	28.7
ViTPose+Transformer	77.8	81.5	95	89.2
RTMPose+TCN	75.2	79.5	42	32.1
Pose-C3D	74.3	79.6	71	55.8
Ours	81.2	86.1	62	51.7

**Table 7** shows that the method proposed in this paper achieves the optimal performance in both pose recognition and damage prediction tasks. Compared with the best result obtained by the baseline model ViTPose+Transformer, it improves the results with gains of 3.4 and 4.6 percentage points, respectively. And it reduces the inference time and the number of parameters by 35% and 42%, respectively.

## 4 Discussion

The multimodal deep learning model reaches an F1-score of 81.2% and AUC of 86.1% on the tasks of posture asymmetry identification and injury risk classification of adolescent table tennis players, thus quantitatively describing the relationship between unilateral force training and structural imbalance. The biomechanical analysis showed that the maximum discrepancy in shoulder rotation Angle was 25.8°, whereas spine lateral flexion Angle was 13° while performing a forehand attack. The area under the receiver-operating characteristic curve was 0.89 for modeling high-risk samples, thereby justifying the model’s ability to detect rare categories. The machine learning models have shown greater efficacy than statistical models in modeling sports injuries (Liu and Wang, 2025). The model of temporal graph encoding together with the graph neural network strongly confirms the potential of deep learning algorithms to solve complicated tasks of causality (Yang et al., 2025).

Essential clarification regarding model scope is warranted. The injury risk classifications reported herein represent expert-based biomechanical screening stratification derived from established clinical thresholds in pediatric sports medicine literature, rather than epidemiologically validated injury incidence forecasts from longitudinal outcome surveillance. This positions the model as a clinical decision support system requiring confirmatory evaluation by qualified practitioners rather than constituting autonomous diagnostic determination.

Beyond algorithmic advances, the observed postural asymmetry patterns warrant interpretation through underlying biomechanical injury mechanisms rooted in table tennis-specific unilateral force generation. Shoulder external rotation asymmetry triggers muscular imbalance cascades through kinetic chain compensation mechanisms wherein dominant-side rotator cuff musculature develops disproportionate strength relative to contralateral stabilizers, creating force couple imbalances at the glenohumeral joint that predispose to impingement syndromes and labral pathology during overhead stroke execution. Repetitive unilateral loading initiates systematic joint load transfer pathway alterations as trunk musculature compensates for inadequate force absorption capacity by recruiting secondary stabilizers across the thoracolumbar junction, progressively overloading spinal erector muscles and inducing asymmetric paraspinal tension manifesting as lateral deviation. The adolescent skeletal development stage amplifies these risk mechanisms through growth-related vulnerability factors including incomplete ossification of apophyseal growth plates at muscle attachment sites particularly vulnerable to repetitive tensile stress, elevated joint laxity from ongoing collagen remodeling reducing passive stability constraints, and muscle strength development lagging behind skeletal growth creating temporary biomechanical mismatches wherein bone lever arms exceed muscular force-generating capacity. Pelvic tilt asymmetries indicate kinetic chain breakdown extending from lower extremity force generation deficits, as inadequate hip extensor strength during stroke preparation necessitates compensatory anterior pelvic rotation transmitting abnormal loading vectors through the lumbar spine and sacroiliac joints.

The injury risk prediction framework operates through a biomechanical causality chain supported by sports medicine literature establishing postural asymmetry as a validated proximal risk factor in the injury etiology pathway. Structural imbalances quantified through asymmetry assessment create aberrant joint loading distributions during repetitive movement execution, initiating progressive microtrauma accumulation in vulnerable musculoskeletal tissues over training exposure periods. Longitudinal investigations in racquet sport populations demonstrate that shoulder rotation asymmetries exceeding clinical thresholds prospectively associate with subsequent rotator cuff pathology development, while trunk lateral deviation patterns predict elevated lumbar injury incidence during competitive seasons. The expert-assessed risk classifications employed in this study translate established biomechanical-injury associations documented in prospective cohort research into screening categories enabling early identification of athletes requiring enhanced monitoring or preventive intervention before tissue damage progresses to symptomatic presentation. This prospective screening paradigm parallels validated clinical instruments including the Functional Movement Screen and Y-Balance Test that successfully stratify injury risk through movement quality assessment despite operating on biomechanical surrogate endpoints rather than direct injury incidence data, reflecting standard practice wherein modifiable risk factors guide preventive interventions in advance of pathological outcomes.

The strategy of multi-modal fusion shows distinct advantages. Comparing to the best single-modality approach, the strategy of three-modal fusion achieves an improvement of about 19 percentage points. The cross-modal attention mechanism shows an improvement of about 5 percentage points over simple splicing. The implementation of multimodal fusion systems incorporating flexible sensors and artificial intelligence for sports injury prevention (Gai, 2025) and the study of wearable technology and motion analysis; (Souaifi et al., 2025) both highlight the role of multi-source fusion for improving prediction results. The result of the visual analysis revealed that the attack involved more procedures based on skeletal geometry, and the serve functioned in reliance on kinematic. The 15–17 years old group performed better than the 12–14 years old group by 3 percentage points, clearly showing the need to begin intervention at this crucial stage.

There is room for further refinement when applying the model. This is because the dataset contains 1,852 test samples, but the fact that labels are derived from expert feedback rather than actual injury data makes it difficult to establish causality. The deep learning model accurately extracts the nonlinear mapping between training load accumulation and occurrences of damage (Sadr et al., 2025), and the real-time monitoring system proves the practicability of the algorithm (Ren et al., 2024). Inference time of 62 ms is better than that of ViTPose with 95 ms, but it is worse than that of RTMPose with 42 ms. The 51.7 million parameter count raises practical deployment considerations for real-world training monitoring applications. Court-side implementation scenarios require evaluation across multiple dimensions including computational hardware requirements wherein inference at 62 ms per frame enables approximately 16 frames per second processing throughput, sufficient for stroke-by-stroke analysis during training sessions yet insufficient for continuous real-time feedback during match play demanding sub-30ms latency. Deployment cost analysis encompasses hardware infrastructure wherein NVIDIA RTX 3090-class GPUs represent substantial capital investment for youth sports facilities, though emerging edge computing platforms such as Jetson AGX Xavier offering 32 TOPS performance at reduced power consumption may enable cost-effective deployment alternatives. Model compression strategies including knowledge distillation to transfer learned representations into lightweight student networks, structured pruning to eliminate redundant parameters while preserving critical feature extraction pathways, and quantization to reduce precision from FP32 to INT8 format could reduce inference latency to sub-40ms ranges with acceptable performance trade-offs. Practical deployment architectures may adopt hybrid processing schemes wherein computationally intensive pose estimation executes on centralized GPU servers while asymmetry quantification and risk classification deploy on edge devices, balancing real-time responsiveness with infrastructure costs. The generalization ability of deep learning for various sports (Zhang et al., 2025; Zheng et al., 2025) reveals that the efficiency of computation needs to be improved by model compression in the future. The recognition accuracy of forehand attack (85.7%) was significantly higher than that of serve (78.9%), suggesting that the training of technical movement diversity should be strengthened.

Multiple fundamental limitations warrant explicit acknowledgment regarding both methodological scope and validation requirements, particularly the absence of external dataset validation and longitudinal injury outcome verification which constrain generalizability claims and predictive validity assertions. The injury risk predictions represent biomechanical risk stratification based on expert-assessed postural deviation patterns requiring prospective validation against actual injury incidence through longitudinal cohort investigations tracking athletes across competitive seasons. While the screening approach follows established sports medicine paradigms wherein biomechanical assessment guides preventive intervention decisions, definitive establishment of predictive validity necessitates calculating sensitivity, specificity, and positive predictive value against observed injury outcomes, calibrating classification thresholds against empirical injury rates, and identifying specific pathology types associated with particular asymmetry patterns. The current framework operates exclusively on postural biomechanical features without incorporating critical training context variables established in sports injury prediction theory including cumulative training volume quantifying repetitive loading exposure, fatigue accumulation reflecting diminished neuromuscular control capacity, and exercise exposure time capturing sport-specific risk accumulation. Multifactorial injury etiology wherein biomechanical predisposition interacts with acute training load spikes and chronic workload-recovery imbalances suggests that comprehensive risk models require integration of wearable sensor data monitoring training metrics alongside postural assessment to capture the temporal dynamics of injury susceptibility. Single-dataset evaluation on TTStroke-21 precludes cross-population generalization testing, with validation needed across diverse athlete populations exhibiting varied technical proficiency from recreational to elite competitive levels, different coaching methodologies producing distinct movement execution patterns, and geographically distributed training facilities reflecting cultural differences in training emphasis and intensity progression. The predominantly methodological and algorithm-driven focus emphasizing network architecture optimization and classification performance enhancement provides limited mechanistic insight into underlying physiological regulation processes including neuromuscular adaptation dynamics during adolescent motor development, motor control maturation affecting movement variability and coordination, and integrative exercise physiology responses governing tissue adaptation versus maladaptation under asymmetric loading conditions.

Beyond these specific methodological constraints, two fundamental validation deficits represent the most critical limitations constraining clinical translation: the absence of external dataset validation and longitudinal injury outcome verification. Evaluation exclusively on TTStroke-21 without independent replication across external cohorts precludes assessment of model robustness to inter-site variability in video acquisition protocols, coaching philosophies influencing movement execution patterns, and population-level differences in injury epidemiology. Cross-dataset validation experiments evaluating performance on geographically distinct athlete cohorts collected under varied experimental conditions would enable quantification of generalization decrements attributable to domain shift, establishing confidence intervals for expected performance in novel deployment contexts. Similarly, the expert-derived risk labels employed for model training and evaluation, while grounded in established biomechanical injury associations documented in sports medicine literature, remain surrogate endpoints requiring empirical confirmation through prospective injury surveillance linking baseline asymmetry classifications to subsequent clinical outcomes. Implementation of longitudinal tracking protocols monitoring injury incidence across multiple competitive seasons stratified by baseline model-predicted risk categories would enable calculation of true sensitivity and specificity metrics, calibration of classification thresholds against observed injury rates, and identification of false negative cases representing biomechanically vulnerable athletes incorrectly classified as low-risk. These validation gaps necessitate cautious interpretation of clinical applicability, positioning the current system as a research prototype demonstrating technical feasibility pending confirmatory real-world validation rather than a deployment-ready clinical instrument with established predictive performance guarantees. Future investigations advancing beyond computational methodology should incorporate longitudinal physiological biomarker assessment including bilateral muscle activation asymmetry quantification via surface electromyography, joint loading distribution measurement through instrumented force platforms, hormonal markers of growth and tissue turnover reflecting systemic adaptation capacity, and neuromuscular fatigue indicators to elucidate physiological mechanisms underlying injury susceptibility beyond static postural evaluation.

## 5 Conclusion

Based on the above analysis, a multimodal deep learning framework for posture asymmetry recognition and sports injury risk prediction in adolescent table tennis players using the RGB sequences, skeletal keypoint trajectory, and kinematic parameters is proposed. The model combines sports-specific adaptations. This involves combining weighted graph convolutional networks with additional edge weights for the racket-holding-arm chain, a four-layer temporal convolutional neural network with a scope that covers an entire stroke cycle, and Focal Loss with dynamic weights. Evaluation on the TTStroke-21 dataset comprising 1,852 test samples demonstrates that the proposed approach achieves 81.2% F1-score for four-class posture asymmetry recognition and 86.1% AUC for three-level injury risk prediction, outperforming the strongest baseline ViTPose+Transformer by 3.4 and 4.6 percentage points while maintaining 62 milliseconds inference time with 51.7 million parameters.

The quantitative analysis reveals the maximum shoulder joint rotation angle asymmetry of 25.8°and the maximum spinal lateral flexion of 13°in the case of forehand drive strokes. On the high-risk dataset, the model achieves the AUC of 0.89 indicating robust minority class sensitivity. Performance stratification reveals forehand drive accuracy of 85.7% substantially exceeding serve actions at 78.9%, while the approximately 3-percentage-point gap between 15–17 and 12–14 age groups underscores the significance of early intervention with high skeletal plasticity. Being able to automate and continuously assess asymmetry of posture has theoretical and practical implications for training observation, since sports coaches and sports physicians can apply individual strategies for contrasting interventions, such as exercising the contralateral limb and core muscle stability before the imbalances result in clinical manifestations of injuries.

## Data Availability

The datasets presented in this study can be found in online repositories. The names of the repository/repositories and accession number(s) can be found in the article/Supplementary Material.
